# Genetic and pharmacological evidence linking CB1R signaling to hippocampal GABAergic dysfunction in ASD mouse model

**DOI:** 10.1016/j.neurot.2026.e01067

**Published:** 2026-09-18

**Authors:** Jingyi Hu, Haoran Wang, Junyu Ren, Tianyu Liu, Yi Jiang, Yilin Zhang, Yuting Zhang, Han Zhang, Caihong Sun, Mingyang Zou

**Affiliations:** a Department of Children’s and Adolescent Health, School of Public Health, Harbin Medical University, Harbin, China; b Heilongjiang Province Key Laboratory of Child Development and Genetic Research, Harbin Medical University, Harbin, China; c Yunnan Institute of Endemic Diseases Control and Prevention (YIEDC), Kunming, China; d Department of Developmental Behavioral Pediatrics, the Sixth Affiliated Hospital of Harbin Medical University, Harbin, China

**Keywords:** CB1R, Autism spectrum disorder (ASD), GABAergic system, Synaptic dysfunction, Conditional knockout

## Abstract

Autism spectrum disorder (ASD) is a neurodevelopmental disorder increasingly linked to disrupted GABAergic inhibition and altered excitatory-inhibitory balance. Cannabinoid receptor 1 (CB1R), enriched on GABAergic interneurons, modulates inhibitory tone. Whether CB1R dysfunction in GABAergic neurons contributes to ASD-like phenotypes remains unclear. This study investigated the role of CB1R in GABAergic regulation and evaluated whether pharmacological CB1R activation could ameliorate GABAergic abnormalities and behavioral deficits in ASD models. We generated conditional CB1R knockout mice in GABAergic neurons (GABA-CB1^−/−^) and performed behavioral and molecular ultrastructural analyses. GABAergic and neuronal alterations were quantified in hippocampus and striatum by Western blotting, immunofluorescence, and transmission electron microscopy. SA-PEG-DSPE nanomicelles were used to deliver the CB1R agonist ACPA or in combination with the GABAA receptor agonist muscimol to valproic-acid (VPA)-exposed mice to assess pharmacological modulation. GABA-CB1^−/−^ mice exhibited social deficits, spatial learning and memory impairments, and repetitive behaviors similar to those of VPA mice. Both models showed increased GAD67 and GABARAP, reduced ABAT, a lower PSD95/gephyrin ratio, a reduced proportion of GAD67+NeuN + neurons, marked dendritic and synaptic disruption in the hippocampus, whereas changes in the striatum were limited. Nanomicelle-mediated ACPA administration ameliorated GABAergic molecular alterations, improved dendritic integrity and ASD-like behaviors, whereas, muscimol co-administration partially attenuated these effects in VPA mice. CB1R deficiency in GABAergic neurons was associated with ASD-like phenotypes accompanied by hippocampal GABAergic dysfunction. Pharmacological CB1R activation ameliorated behavioral and molecular abnormalities, while modulation of GABAAR partially attenuated these effects. These findings support further investigation of CB1R as a potential therapeutic target for ASD.

## Introduction

Autism spectrum disorder (ASD) is a complex neurodevelopmental disorder characterized by core symptoms of social impairments and repetitive stereotyped behaviors, frequently accompanied by cognitive defects [1]. The pathophysiology of ASD is thought to be associated with the imbalance between excitatory and inhibitory (E/I) signaling within the neural circuits [2,3], especially, gamma-aminobutyrate (GABA), as the major inhibitory neurotransmitter, its function plays a pivotal role in maintaining E/I balance [4]. The postmortem studies have discovered the structural and functional changes in GABAergic inhibitory circuits in individuals with ASD [5]. Humans and rodent data further implicated that elevated GABA concentration has been observed in the peripheral blood and brain tissue of ASD patients, which correlated with the severity of ASD symptoms [6,7]. These alternations coincide with dysfunction of GABA synthetic enzymes glutamate decarboxylase isoforms (GAD)65 and GAD67, GABA receptors, transporters vesicular GABA transporter (VGAT) and metabolic enzymes such as 4-aminobutyrate aminotransferase (ABAT), suggesting broad disturbance of GABA homeostasis in ASD [[8], [9], [10], [11], [12]]. Importantly, pharmacological modulation of GABA receptor has been shown to ameliorate core behavioral deficits and restore hippocampal dendritic abnormalities in ASD models [13].

The cannabinoid receptor type 1 (CB1R) is a G protein-coupled receptor abundantly expressed at GABAergic synapses throughout the hippocampus, prefrontal cortex and striatum [14]. Functional crosstalk between GABAergic signaling and CB1R has been reported, with CB1R regulating inhibitory homeostasis by modulating GABA synthesis, vesicular release, and signal transduction [15,16]. CB1R signaling also influences the migration, differentiation and maturation of GABAergic neurons [17,18] and GABAergic synaptic plasticity during development [15]. Given this key role in the regulation of inhibitory network integrity, CB1R has emerged as an interesting candidate for ASD development. Accumulating evidence supported a close association between CB1R dysfunction and ASD. Our previous study demonstrated that CB1R knockout mice exhibited classic ASD-like behaviors, including social deficits, restricted and repetitive behaviors and cognitive impairments, alongside synaptic dysfunction persisting into adulthood. Conversely, CB1R activation mitigated synaptic abnormalities, as evidenced by improvements in neuronal complexity, spine density, dendritic integrity, synaptic protein expression, and reductions in neuronal damage [19]. Parallel studies confirmed that pharmacological or genetic manipulations of CB1R influenced ASD-like behavioral phenotypes across multiple species [20,21]. Nevertheless, whether and how CB1R-mediated GABAergic regulation contributes to ASD-like phenotypes remains largely unknown.

Therefore, we hypothesized that altered CB1R signaling is associated with GABAergic alterations and ASD-like phenotypes, and that pharmacological modulation of CB1R may influence these abnormalities in ASD models. Unlike previous studies that primarily relied on global CB1R knockout or systemic pharmacological manipulation, the present study employed GABAergic neuron-specific CB1R deletion and systematically assessed GABAergic alterations at molecular, cellular, and ultrastructural levels across multiple brain regions, with the well-established VPA-induced ASD model as a positive reference. We further employed nanomicelle-mediated pharmacological intervention to explore the relationship between CB1R activation and GABAergic signaling in an ASD-relevant context. This study provides new insights into the association between CB1R signaling and GABAergic alterations in ASD-relevant pathology and supports further investigation of CB1R as a potential therapeutic target.

## Materials and methods

### Experimental design

This study was conducted in two sequential parts (Fig. 1). Part 1 (functional correlation) determined whether selective deletion of CB1R in forebrain GABAergic neurons is sufficient to produce ASD-like phenotypes. Behavioral, molecular, and ultrastructural outcomes were assessed in GABA-CB1R conditional knockout (CKO) mice and their WT littermates, with the prenatal valproic acid (VPA) model included as a well-established ASD reference (positive control) for benchmarking key ASD-relevant readouts. Part 2 (pharmacological rescue) used VPA-exposed offspring to test whether nanomicelle-mediated CB1R activation can reverse ASD-like abnormalities and to probe the involvement of GABAA-related signaling, by comparing nanomicelle-delivered ACPA (CB1R agonist) treatment with ACPA plus muscimol (GABA receptor agonist) co-administration. For each part animals were not reused across experiments.

**Fig. 1:**
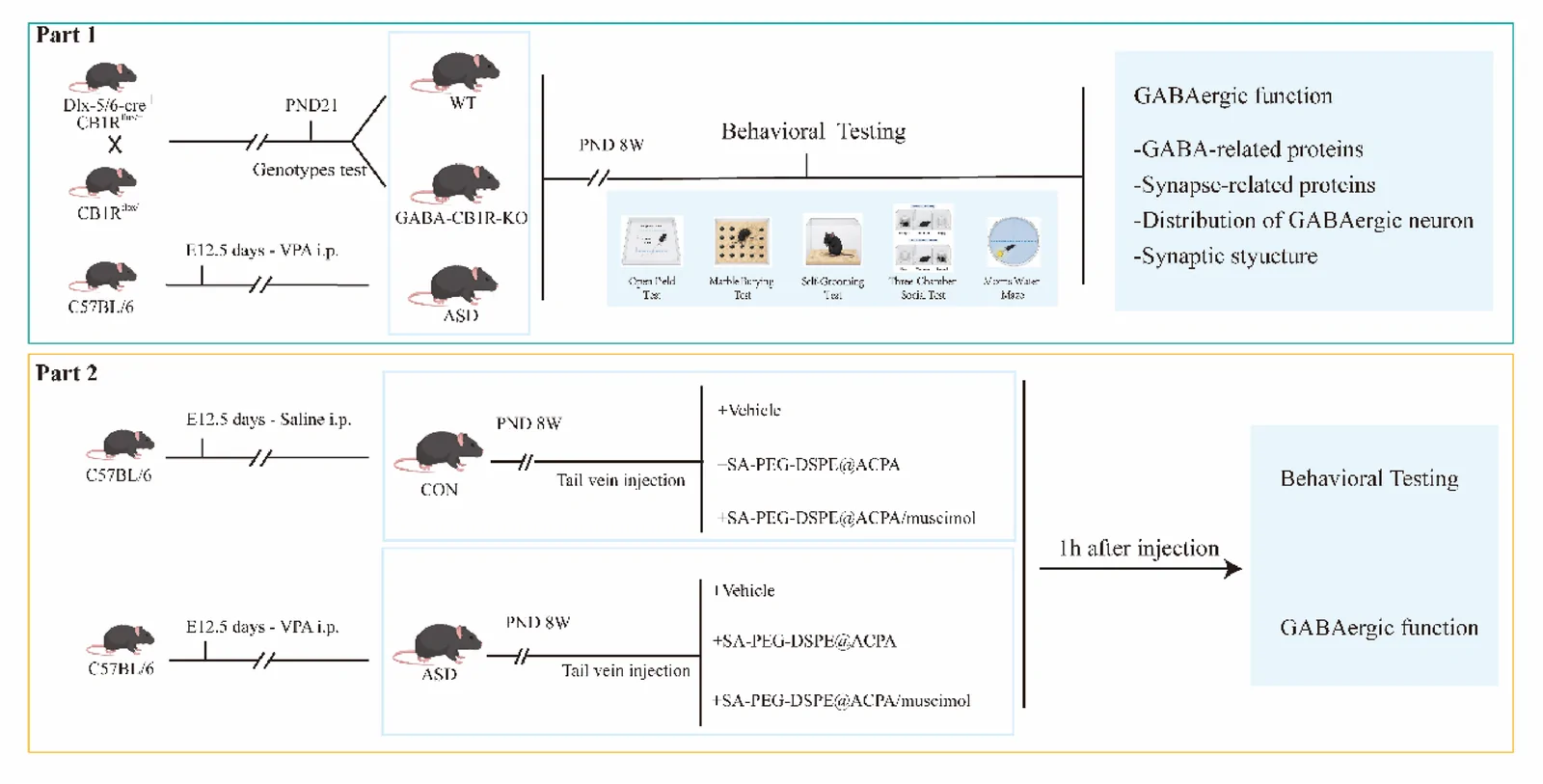
Flowchart for the experiment.

### Mice

C57BL/6 mice and Cnr1^flox/flox^ mice (harboring loxP-flanked CB1R) were purchased from Beijing Weitong Lihua Laboratory Animal Technology Co., Ltd. (Beijing, China) and GemPharmatech Co. Ltd. (Nanjing, China), respectively. Dlx5/6-Cremice (expressing Cre recombinase specifically in forebrain GABAergic neurons) with a C57BL/6 background were generously provided by Professor Yang Zhenggang (Fudan University). Homozygous Cnr1^flox/flox^ mice were crossed with Dlx5/6-Cre transgenic mice to obtain Dlx5/6-Cre^+^;Cnr1^flox/flox^ conditional knockout mice (CKO; GABA-CB1^−/−^), and littermates Dlx5/6-Cre^-^;Cnr1^flox/flox^ (WT) (Fig. 2A). All lines were maintained on a C57BL/6 genetic background. Genotyping was performed via polymerase chain reaction (PCR) analysis of tail DNA using specific primers (primer in supplemental materials).

**Fig. 1:**
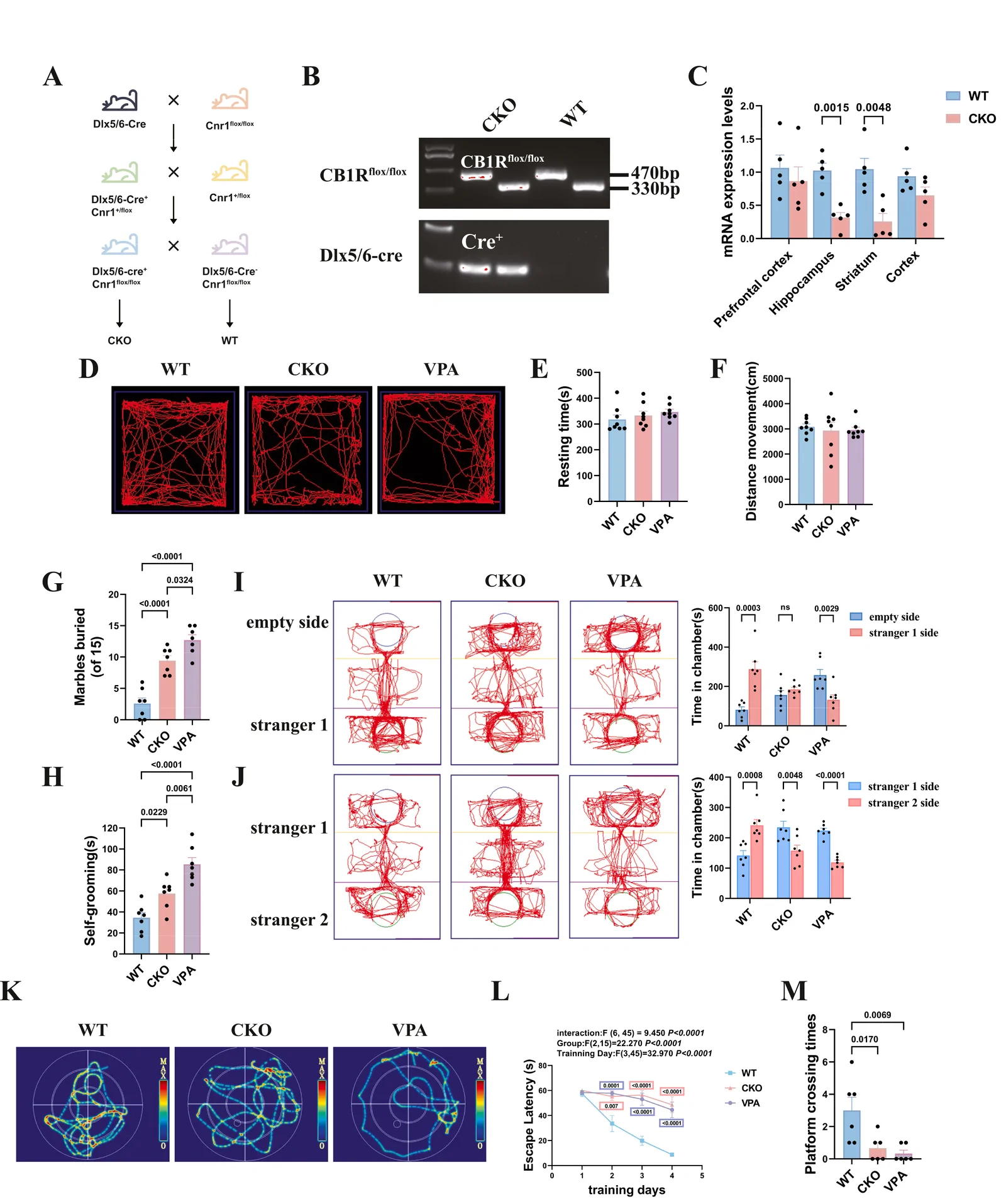
**GABA-CB1^−/−^mice displayed social deficits, repetitive behavior, impaired spatial learning, and memory.** (A) Breeding strategy for generating GABAergic CB1R conditional knockout (CKO) and wild-type (WT) mice. (B) Representative PCR genotyping results of CKO and WT mice. (C) Relative mRNA expression levels of CB1R in the prefrontal cortex, hippocampus, striatum, and cortex of CKO mice compared to WT controls. Unpaired student *t*-test. (D–F) No significant differences among the three groups were observed in the open field test for resting time and total distance movement. One-way ANOVA. (G and H) VPA and CKO mice exhibited increased repetitive behaviors, including prolonged grooming duration and higher marble-burying counts. One-way ANOVA. (I) In the sociability test, VPA mice showed a significant preference for interacting with empty side relative to WT mice, but CKO did not significant preference for empty side and stranger 1. Unpaired student *t*-test. (J) In the social preference test, WT mice preferred stranger 2, while VPA and CKO mice preferred stranger 1. Unpaired student *t*-test. (K–M) Morris water maze performance: compared to WT mice, CKO and VPA mice showed longer escape latency across training days and significantly fewer platform crossings during the probe trial, indicating impaired spatial learning and memory. Two-way ANOVA. All data are presented as means ± SEM, n = 5–8 per group; One-way ANOVA multiple comparisons with Turkey and two-way ANOVA with Sidak post hoc test.

ASD mice model was established by prenatal valproic acid (VPA) exposure in accordance with previous reported protocols [22]. Briefly, pregnant females received a single intraperitoneal (i.p.) injection of VPA (Sigma Aldrich, St. Louis, MO, USA) at 600 mg/kg dissolved in 0.9% saline on embryonic day 12.5 (E12.5). Control pregnant animals were administered saline only. Pregnant mice were housed separately and allowed to care for their offspring. Offspring were weaned on postnatal day (PND) 21, housed 4–5 per cage under standard conditions.

Mice were maintained under standard specific pathogen-free conditions in a controlled environment (temperature: 22 ± 2 °C; humidity: 50 ± 10%; 12-h light/dark cycle, lights on 7:00) with ad libitum food and water. Only male offspring were used because males exhibit greater susceptibility and more consistent behavioral responses in the VPA-induced ASD model. Sample sizes were determined based on previous studies employing similar experimental designs and outcome measures, together with pilot observations from our laboratory. Accordingly, the first phase requires a total of 18–24 animals (i.e., 3 groups), and the second phase requires a total of 36–48 animals (i.e., 6 groups). Inclusion/exclusion criteria were predefined before experiment. Animals showing illness, surgical failure, or premature death were excluded. no animals met these exclusion criteria during the study. Animals were randomly allocated (random number table), with testing order and cage position randomized to minimize confounding. All experimenters were blnded to genotype and treatment throughout allocation, experimentation. All procedures and protocols were reviewed and approved by the Harbin Medical University Animal Care and Use Committee (HMUIRB20200007).

### Preparation of SA-PEG-DSPE nanomicelles

Sialic acid (SA)-polyethyleneglycol (PEG)-distearoyl phosphatidylethanolamine (DSPE) nanomicelles was kindly gifted by Changmei Zhang (Daqing Campus, Harbin Medical University) for subsequent nanomicelle-mediated interventions. SA-PEG-DSPE@ACPA nanomicelles, incorporating CB1R agonist ACPA (MedChemExpress, Monmouth Junction, NJ, USA), and SA-PEG-DSPE@muscimol nanomicelles, incorporating GABA receptor agonist muscimol (Sigma Aldrich, St. Louis, MO, USA), were prepared for the following studies.

***In Vivo Evaluation****:* To evaluate the in vivo targeting efficacy of the nanomicelles, SA-PEG-DSPE nanomicelles encapsulating the near-infrared dye DiR (1 mg/kg; Macklin, Shanghai, China) were intravenously injected into C57BL/6J mice. Fluorescence biodistribution was assessed using an in vivo imaging system (Carestream In Vivo FX, Bruker, Madison, WI, USA). Optical and fluorescence images were captured immediately post-injection at excitation and emission wavelengths of 720 and 790 nm, respectively.

***In Vitro Evaluation****:* Highly differentiated PC12 cells (rat adrenal pheochromocytoma cell line; Procell Life Technology, Wuhan, China) were cultured in DMEM/F12 medium supplemented with 10% fetal bovine serum (FBS), 1% penicillin-streptomycin, and 0.1% sodium pyruvate. Cells were maintained at 37 °C in a humidified incubator containing 5% CO_2_ and passaged every 2–3 days. For cellular uptake studies, PC12 cells were seeded in 6-well plates and cultured until reaching 70–80% confluence. SA-PEG-DSPE nanomicelles encapsulating DiO fluorescent dye or an equivalent amount of free DiO were added to the medium and incubated for 4 h. After incubation, cells were washed with phosphate-buffered saline (PBS) to remove excess dye, collected into centrifuge tubes, resuspended, and prepared as single-cell suspensions. Nanomicelles cellular uptake was quantified using flow cytometry (Beckman Coulter, Brea, CA, USA).

***Nanoparticle Characterization:*** To prepare drug-loaded nanomicelles, ACPA, muscimol, and SA-PEG-DSPE were dissolved separately in chloroform at final concentrations of 10 mg/mL, 5 mg/mL, and 0.5 mg/mL, respectively. Solutions were vortexed briefly to ensure complete dissolution. The ACPA or muscimol solution was then added dropwise to the SA-PEG-DSPE solution under continuous vortexing. Following thorough mixing, the chloroform was allowed to evaporate completely under reduced pressure. The resulting film was rehydrated with 0.9% sodium chloride (NaCl) solution and sonicated to obtain a homogenous nanoparticle suspension.

For morphological assessment, 0.5 mg/mL of nanoparticle solution was placed on a carbon-coated copper grid, blotted with filter paper, air-dried, and stained with 2% phosphotungstic acid. Samples were examined under a transmission electron microscope (TEM; H-600, Hitachi, Tokyo, Japan). Particle size, polydispersity index, and zeta potential were measured using a dynamic light scattering analyzer (Malvern Instruments, UK) with 3 mL of nanoparticle suspension in a standard cuvette.

***Hemolysis assay:*** The hemocompatibility of SA-PEG-DSPE nanomicelles was evaluated using a red blood cell (RBC) hemolysis assay. Arterial blood was collected from C57BL/6 mice and prepared as a 2% RBC suspension. Nanomicelles solutions at various concentrations (0.005, 0.01, 0.03, 0.05, 0.1, 0.3, 0.5, and 1 mg/mL) were mixed with equal volumes of RBC suspension. Double-distilled water (ddH_2_O) and 0.9% saline were used as positive and negative controls, respectively. After 1 h of incubation at room temperature, samples were centrifuged, and the supernatants were transferred to a 96-well plate. Absorbance at 540 nm was measured using a microplate reader (Synergy H1, Agilent Technologies). The hemolysis rate (%) was calculated using the formula: Hemolysis rate (%) = (OD_Experimental_ − OD_negative_)/(OD_positive_ − OD_negative_) × 100%. A hemolysis rate >5% was considered indicative of significant hemolysis.

***Cytotoxicity Assay (MTT):*** The cytotoxicity of SA-PEG-DSPE nanomicelles was assessed using the MTT assay. PC12 cells were seeded in 96-well plates and incubated overnight. Cells were treated with various concentrations of nanomicelles (0.005, 0.01, 0.03, 0.05, 0.1, 0.3, and 0.5 mg/mL) for 24 h. After treatment, the culture medium was removed, and 100 μL of DMSO was added to dissolve formazan crystals. Absorbance at 490 nm was measured using a microplate reader (Synergy H1, Agilent Technologies). Cell viability was calculated using the formula: Cell viability (%) = (OD_Experimental_ − OD_blank_)/(OD_control_ − OD_blank_) × 100%.

***Encapsulation Efficiency (EE) and Loading Efficiency (LE) assay:*** EE and LE were determined to evaluate the drug-loading capacity and stability of nanomicelles. Serial dilution of SA-PEG-DSPE@ACPA and SA-PEG-DSPE@muscimol were dissolved in anhydrous ethanol, respectively, and absorbance at 350 nm was measured using a UV-5100B spectrometer to obtain standard curves. Following dilution of SA-PEG-DSPE nanomicelles, OD values were determined and applied to the corresponding standard curve for drug concentration quantification. EE and LE were calculated according to the following equations:

$$\text{EE}\;\left(\%,w/w\right)=\frac{\text{ACPA}/\text{muscimol}\;\text{encapsulated}\;\text{content}}{\text{ACPA}/\text{muscimol}\;\text{total}\;\text{content}}\text{;}$$

$$\text{LE}\;\left(\%,w/w\right)=\frac{\text{ACPA}/\text{muscimol}\;\text{encapsulated}\;\text{content}}{\text{ACPA}/(\text{muscimol}\;\text{encapsulated}\;c\text{ontent}+m\text{icelles}\;\text{content}}$$

***Dosage selection:*** For in vivo intervention studies, C57BL/6J male mice (8 weeks old) were randomly assigned to vehicle control or treatment groups. Mice in the control group received an intravenous injection (tail vein, i.v.) of 0.9% NaCl. Treatment groups were administered SA-PEG-DSPE@ACPA or SA-PEG-DSPE@muscimol nanomicelles at three dosage levels: 0.1 mg/kg, 0.3 mg/kg, and 1 mg/kg. Based on dose–response analyses (see Fig. S1C and D), 0.3 mg/kg ACPA-loaded nanomicelles was selected as the effective intervention dose based on its significant modulation of CB1R protein expression. Similarly, the 1 mg/kg muscimol-loaded nanomicelles was chosen for intervention based on its robust impact on the expression levels of GAD67 and GABARAP proteins.

### Drug administration

VPA-exposed offspring (VPA group) and their corresponding control mice (CON group) were administered 0.3 mg/kg SA-PEG-DSPE@ACPA nanomicelles and 1 mg/kg SA-PEG-DSPE@muscimol via intravenous injection (tail vein, i.v.). Male offspring aged at 8 weeks, derived from different litters, were randomly assigned to the following experimental groups: vehicle group: CON or VPA mice were injected with 0.9% NaCl; ACPA group: CON or VPA mice were injected with 0.3 mg/kg SA-PEG-DSPE@ACPA nanomicelles; ACPA + muscimol group: CON or VPA mice were injected with 0.3 mg/kg SA-PEG-DSPE@ACPA and 1 mg/kg SA-PEG-DSPE@muscimol, with a 30-min interval.

After the treatment, behavioral experiments were performed. All mice were then sacrificed, and the brain tissues were homogenized and fixed for RT-PCR, western blotting and immunofluorescent staining and transmission electron microscope analysis, respectively.

### Behavioral testing

All behavioral tests were conducted during the light phase between 09:00 and 17:00. Testing sessions were recorded using high-definition video cameras, and behavioral analyses were performed using the SMART v3.0 software system (Panlab, Barcelona, Spain). The testing apparatus was cleaned with 75% ethanol between trials to eliminate residual olfactory cues. Observers blnded to group assignments analyzed all behavioral data to minimize bias. The general order of behavioral testing was open field test, marble burying test, self-grooming test, three-chamber test, and Morris water maze test. No animals were single-housed or socially isolated prior to behavioral testing.

***Open field test:*** Spontaneous locomotor activity was assessed utilizing an open field test. The open-field apparatus consisted of a charcoal gray plastic box (450 × 450 × 450 mm) with a top opening. Mice were habituated to the testing arena for 5 min prior to behavioral recording to minimize stress-induced behavioral variability. Subsequently, each mouse was placed in the center of the arena and allowed to explore freely for 10 min. The total distance movement, resting time, and movement trajectory were recorded.

***Marble burying test:*** Mice were individually placed in a standardized white plastic box (320 × 202 × 135 mm) containing a 4 cm-deep layer of compacted wood chip bedding. Fifteen black marbles (12 mm diameter) were arranged in a 3 × 5 matrix on the surface. The number of marbles buried (defined as covered by bedding by more than two-thirds) over a 10-min period was recorded to assess repetitive and stereotyped behavior.

***Self-grooming test:*** Mice was placed in an identical white plastic box (320 × 202 × 135 mm) for 5-min acclimation period, followed by a 10-min observation period during which spontaneous self-grooming behavior was recorded. The cumulative duration of grooming was quantified. The environment was kept quiet throughout the procedure to minimize external disturbances.

***Three-chamber test:*** This test was primarily used to evaluate social behavior. The test comprised two stages. Mice were first acclimated by being placed in the central chamber for 5 min. The test included two 10-min successive tests: 1) Sociability test: Animals were briefly confined to the central chamber while an unfamiliar mouse (labeled as stranger 1) was confined in a small wire cage which was placed in one of the outer chambers. An identical empty wire cage was placed in the other chamber. The test mouse’s time spent in each chamber was recorded. 2) Social preference test: A novel unfamiliar mouse (labeled as stranger 2) was then placed in the empty cage. The test mouse’s total distance traveled and time spent exploring Stranger 1 and Stranger 2 were recorded. All stimulus mice were age- and sex-matched and unfamiliar to the test subjects, with no prior physical contact.

***Morris water maze:*** This test was used to assess learning and spatial memory. Morris water maze consisting of a circular pool (diameter: 1.2 m; height: 0.6–0.8 m) filled with opaque water (22–25 °C). A submerged escape platform (diameter: 10 cm) was placed 1–2 cm below the water surface in one quadrant. The test consisted of two stages: 1) Training sessions (Days 1–4): Mice received two training sessions daily from randomized start positions. Each session lasted a maximum of 60 s. The escape latency, swimming trajectory and swim path length were recorded. 2) Spatial Probe Test (Day 5): The platform was removed. The number of times that mice crossed the circular area of the original platform within 60 s was recorded as an index of spatial memory. Swim speed was calculated as the swim path length divided by the escape latency: Speed (mm/s) = Swim path length/Latency. The swim path length is defined as the total distance swum from the start point to the hidden platform, and the latency as the time taken to reach it. This parameter served as a measure of basal locomotor and visuomotor function, enabling the exclusion of potential confounding effects of motor performance on spatial learning and memory outcomes.

### Western blotting

After behavioral testing, mice were anesthetized with 10% chloral hydrate (1 mL/kg) and sacrificed. The prefrontal cortex, hippocampus, striatum, and cortical tissues were dissected on ice and stored at −80 °C.

Protein was extracted and the concentration was quantified with a BCA protein assay kit (Beyotime). After separation by 10% SDS-PAGE and transfer to polyvinylidene difluoride membranes, the blots were incubated with primary antibodies [CB1R 1:1000, postsynaptic density protein 95 (PSD95) 1:4000, GAD67 1:10000, GAD65 1:10000, vesicular GABA transporter (VGAT) 1:800, 4-Aminobutyrate aminotransferase (ABAT) 1:1000, vesicular glutamate transporter 1 (VGLUT1) 1:4000, GABA type A receptor-associated protein (GABARAP) 1:2000, synapsin-1 (SYN1) 1:5000, gephyrin (Gep) 1:2000, GAPDH 1:50000] and corresponding secondary antibodies. Protein bands were visualized using a ChemiDoc XRS + system (Bio-Rad Laboratories) with an enhanced chemiluminescence detection kit (Beyotime). The relative expression levels of protein compared to GAPDH were calculated using Image J/Fiji (National Institutes of Health, Bethesda, MD, USA). Measurements were obtained from triplicate samples.

### RNA Extraction, reverse transcription, and qRT-PCR

Total RNA was extracted using the Trizol reagent (Invitrogen, Thermo Fisher Scientific, Waltham, MA, USA) following the manufacturer’s protocol. After reverse transcription to cDNA using a thermal cycler (T-100, Bio-Rad Laboratories, Hercules, CA, USA), a quantitative PCR assay was performed on the Light Cycler 480 system (Roche Applied Science, Indianapolis, IN, USA) with SYBR Green PCR Master Mix (Applied Biosystems Inc., Foster City, CA, USA). The primers for qRT-PCR were as supplementary materials. GAPDH was used to normalize gene expression data. Fold-change expression was calculated by the 2^−ΔΔCT^ method.

### Immunofluorescence

For histological studies, mice were deeply anesthetized and transcardially perfused with PBS and 4% paraformaldehyde (PFA). Brains were post-fixed, cryoprotected sucrose, and embedded in OCT compound. Coronal brain sections (6–8 μm in thickness) were cut on a Leica CM1950 cryostat (Leica, Wetzlar, Germany), thaw-mounted onto glass slides, air-dried, and stored at −20 °C until processing for immunofluorescence. Brain sections were blocked with 10% goat serum for 1 h and subsequently incubated with primary antibodies (GAD67 1:200, NeuN 1:500, MAP2 1:100, CB1R 1:100) at 4 °C overnight, followed by secondary antibody for 1 h. After nuclear staining with DAPI, the sections were photographed (20 × lens) under a fluorescence microscope (Zeiss, Germany). Quantitative assessment in specific regions (hippocampal CA1 and CA3 and the striatum) was conducted on the acquired images using Image J Fiji software (National Institutes of Health, Bethesda, MD, USA). GAD67^+^/NeuN^+^ double-positive cells were identified using standardized thresholding. The percentage was calculated as (GAD67^+^/NeuN^+^ cells/total NeuN^+^ cells) × 100%. For MAP2 analyses, fluorescence images were thresholded, and the positive area fraction (%) was calculated as the marker-positive area divided by the total analyzed area. Threshold settings were kept identical for all sections within each marker to ensure comparability across groups.

### Transmission electron microscopy

Following deep anesthesia, the hippocampus and striatum were isolated and sectioned into ∼1 mm^3^ tissue blocks. Samples were immediately fixed in 2.5% glutaraldehyde at 4 °C for ≥2 h, rinsed in 0.1 M phosphate buffer (pH 7.4) and post-fixed with 1% osmium tetroxide at 4 °C for 1–2 h. Following dehydrated through a graded ethanol series and acetone, tissues were embedded in epoxy resin and polymerized. Semi-thin sections (2 μm) stained with toluidine blue were used for target regions localization. Subsequently, ultrathin sections (70–90 nm) were collected on copper grids and double-stained with2% uranyl acetate and lead citrate. Ultrastructural images were acquired using a transmission electron microscope (Hitachi, Japan, HT7700) under appropriate settings to evaluate neuronal and synaptic components in the hippocampus and striatum.

### Statistical analyses

Data were presented as means ± S.E.M. All statistical analyses were performed with GraphPad Prism 8.0 (GraphPad Software, CA, USA). Unpaired student t-tests, one-way ANOVA (with Tukey or Sidak post hoc tests), or two-way ANOVA were used for comparisons between two groups, among multiple groups. Statistical significance was defined at *P* < 0.05. Detailed statistical information for all analyses is summarized in Supplementary Table 1.

## Results

### Generation and characterization of transgenic animal models

**Fig. 2:**
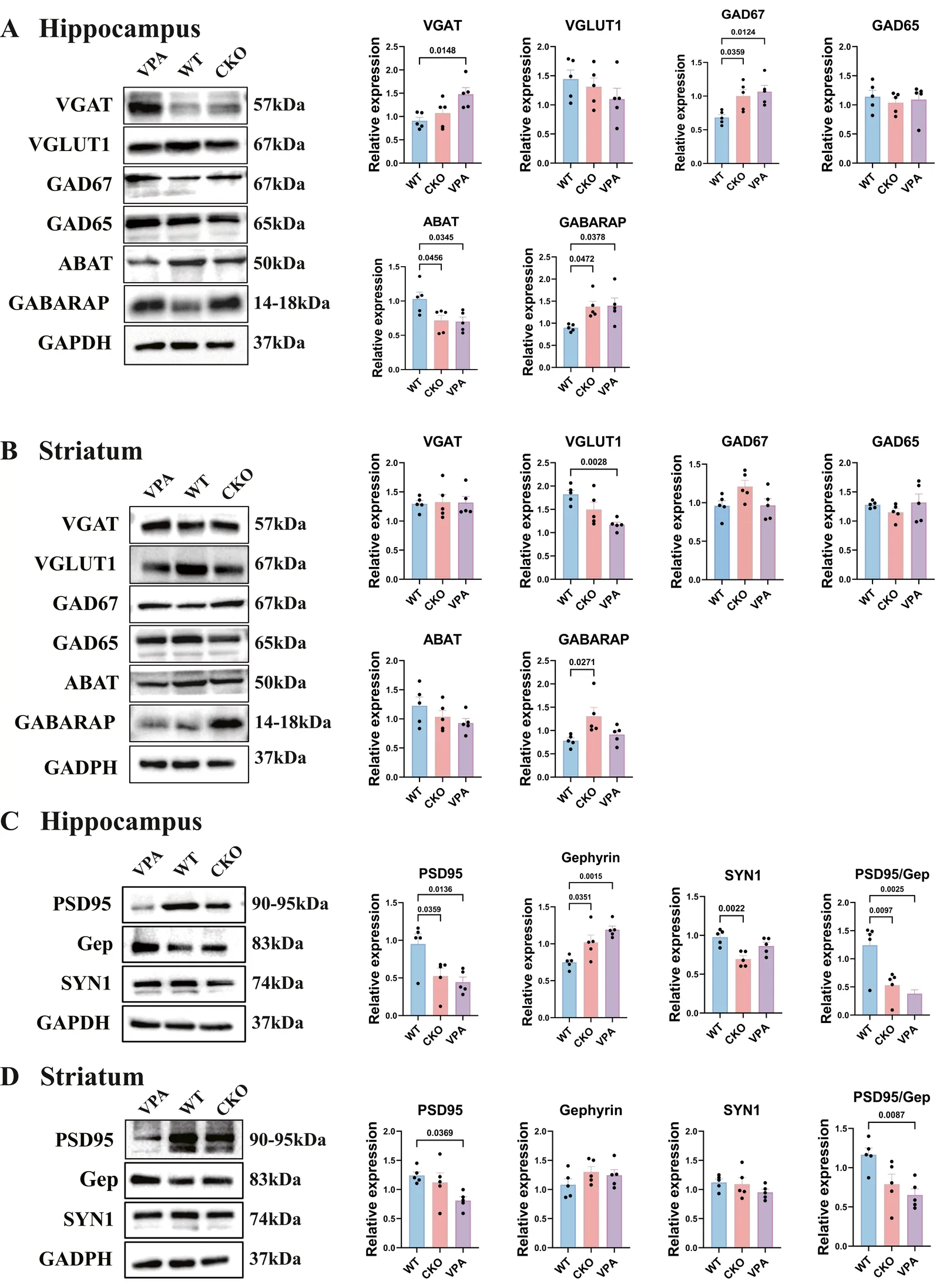
**Altered expression of GABAergic and synaptic proteins in the hippocampus and striatum of WT, CKO, and VPA mice.** (A) Representative immunoblots and quantification of GABAergic-related proteins, including VGAT, GABARAP, GAD67, ABAT, GAD65, and VGLUT1 in the hippocampus. VPA and CKO mice exhibited significant upregulation of GAD67 and GABARAP, and downregulation of ABAT, relative to WT controls. One-way ANOVA. (B) Representative immunoblots and quantification of GABAergic-related proteins in the striatum. CKO mice showed elevated GABARAP levels, while other markers remained unchanged. One-way ANOVA. (C) Representative immunoblots and quantification of synaptic proteins, including PSD95, Gephyrin, SYN1, PSD95/Gep in the hippocampus. Both VPA and CKO mice displayed reduced PSD95, elevated Gephyrin, and decreased PSD95/Gep ratio. CKO mice additionally showed decreased SYN1 expression. One-way ANOVA. (D) Representative immunoblots and quantification of synaptic proteins in the striatum. VPA mice exhibited reduced PSD95 and PSD95/Gep ratio, with no significant changes in CKO mice compared to WT. One-way ANOVA. All data are presented as means ± SEM, n = 5 per group; multiple comparisons with Turkey post hoc test.

Representative genotyping results of GABA-CB1^−/−^ (hereafter referred to as CKO) and their WT were shown in Fig. 2B. Although CKO mice were viable and fertile, their body weights were lower than WT mice from P21 to adulthood (Fig. S1A). Additionally, the gross brain size of CKO mice appeared smaller than that of WT mice (Fig. S1B). Quantitative analysis of CB1R expression in the brain tissue showed that mRNA levels of CB1R in the hippocampus and striatum of CKO mice were significantly decreased by approximately 50% compared to their WT controls, while no significant difference in the cortex (Fig. 2C). (Prefrontal cortex, t_8_ = 0.694, *P* = 0.256. Hippocampus, t_8_ = 5.290, *P* = 0.002. Striatum, t_8_ = 3.869, *P* = 0.005. Cortex, t_8_ = 1.691, *P* = 0.087). Furthermore, double immunofluorescence staining demonstrated the colocalization of CB1R with the GABAergic neuronal marker GAD67 in the hippocampal CA1 and CA3 regions. (Fig. S2).

### GABA-CB1^−/−^ mice displaying ASD-like behavioral phenotypes

In the open field test, no significant differences were observed among VPA, WT, and CKO mice in terms of resting time (F_2,21_ = 0.905, *P* = 0.420) or total distance movement (F_2, 21_ = 0.148, *P* = 0.864), suggesting that general locomotor activity was not affected (Fig. 2D–F). In the self-grooming test and the marble burying test, VPA mice exhibited more stereotypic behaviors than WT mice, as evidenced by increased grooming time (F_2,18_ = 21.12, *P* < 0.0001) and a greater number of buried marbles (F_2,18_ = 37.98, *P* < 0.0001). Similarly, CKO mice showed comparable increases in these repetitive behaviors relative to WT. (Fig. 2G and H). In both phases of the three-chamber social test, no significant differences in total distance traveled were observed among groups (Fig. S3A–B). Results of the three-chamber test demonstrated that VPA-exposed offspring, a well-established model of ASD, exhibited significant social deficits. Compared to WT mice, VPA mice spent more time in the empty chamber than in the mouse-containing chamber, also spent more time in the familiar mice than in the stranger. Notably, CKO mice exhibited significant social impairment relative to WT controls, failing to distinguish between familiar and newly introduced conspecifics (Fig. 2I–J) (Phase1, WT, t_12_ = 5.107, *P* = 0.0003. CKO, t_12_ = 1.104, *P* = 0.098. VPA, t_12_ = 3.352, *P* = 0.003. Phase 2, WT, t_12_ = 4.059, *P* = 0.0008. CKO, t_12_ = 2.862, *P* = 0.005. VPA, t_12_ = 9.809, *P* < 0.0001.). In the Morris test, two-way ANOVA analyses revealed significant interaction effects of group and training duration on escape latency. WT mice demonstrated progressive reductions in escape latency over training sessions, whereas, VPA and CKO mice showed significantly longer escape latencies throughout training (Fig. 2L). Swim speed did not differ significantly among the three groups on any training day (Fig. S4A). In contrast, two-way ANOVA revealed a significant interaction between group and training day for swim path length. Swim path length progressively decreased across training days in all groups but remained significantly longer in VPA and CKO mice than in WT mice (Fig. S4B). During the probe test, both VPA and CKO mice crossed the previous platform location significantly fewer times than WT mice (Fig. 2M) (F_2,15_ = 7.703, *P* = 0.005). Swim speed remained comparable among the three groups (Fig. S4C). CKO mice showed a significantly longer swim path length than WT mice, whereas the difference between VPA and WT mice did not reach statistical significance (Fig. S4D). Taken together, these findings confirmed that VPA-exposed mice, serving as a positive control, exhibit hallmark behavioral deficits consistent with ASD. Importantly, CKO mice also showed social impairments, increased stereotypy, and cognitive dysfunction, with an overall pattern broadly consistent with the VPA phenotype, suggesting that CB1R deletion in GABAergic neurons leads to an ASD-like phenotype.

### Abnormal GABAergic function in the hippocampus of *GABA-CB1*^*−/−*^ mice

**Fig. 3:**
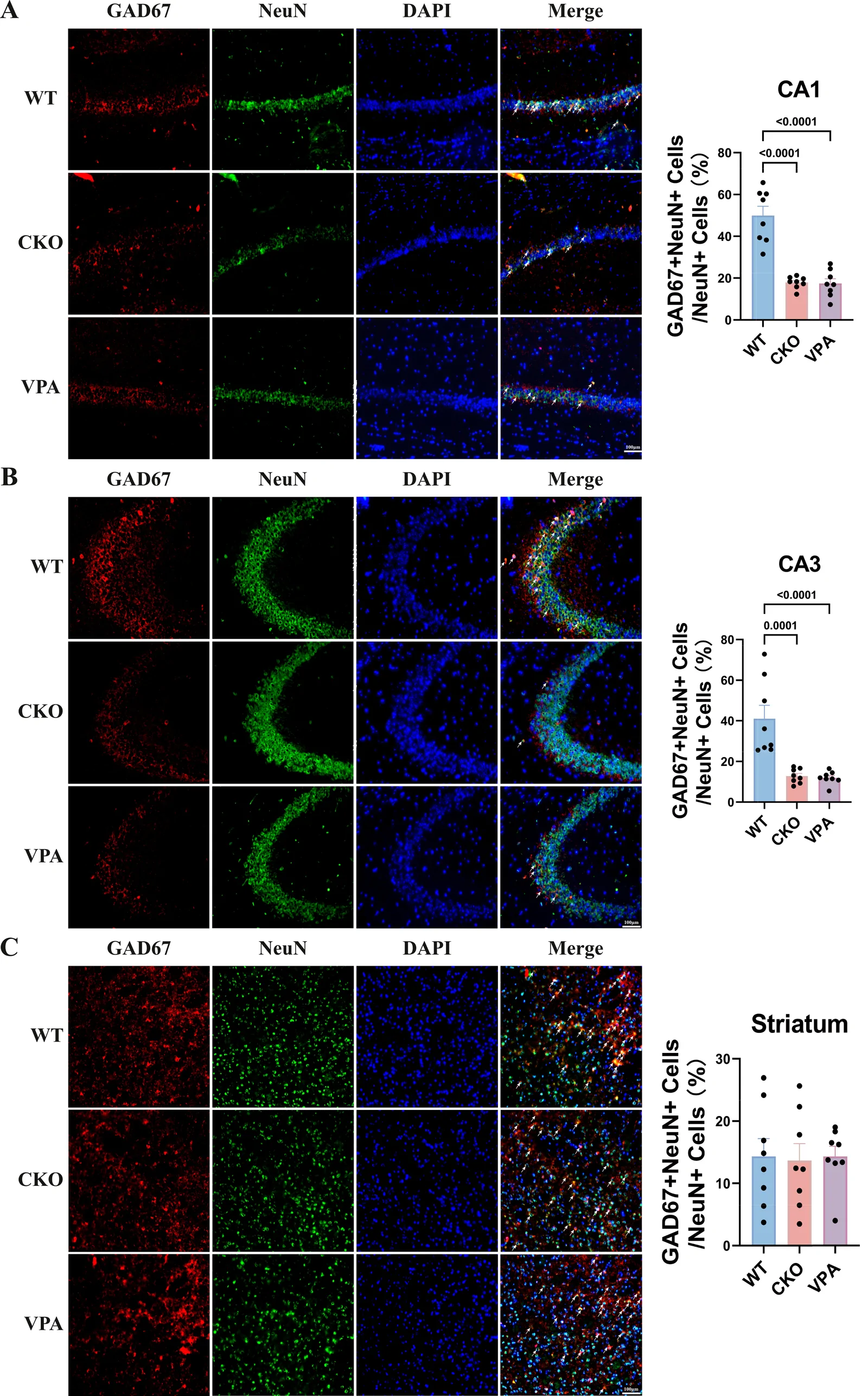
**Distribution of GABAergic neurons in hippocampus and Striatum of WT, CKO, and VPA mice.** (A–C) Representative images showing co-localization of GAD67 (red) and NeuN (green) in hippocampal CA1, CA3, and striatum regions. Nuclei were counterstained with DAPI (blue). Scale bar, 100 μm. Quantification revealed a significant reduction in GAD67^+^NeuN^+^ double-positive neurons in the CA1 and CA3 regions of VPA and CKO mice compared to WT controls. No significant alterations were observed among groups in the striatum. All data are presented as means ± SEM, n = 4 per group, 2 slices; one-way ANOVA with Turkey post hoc test is used.

**Fig. 4:**
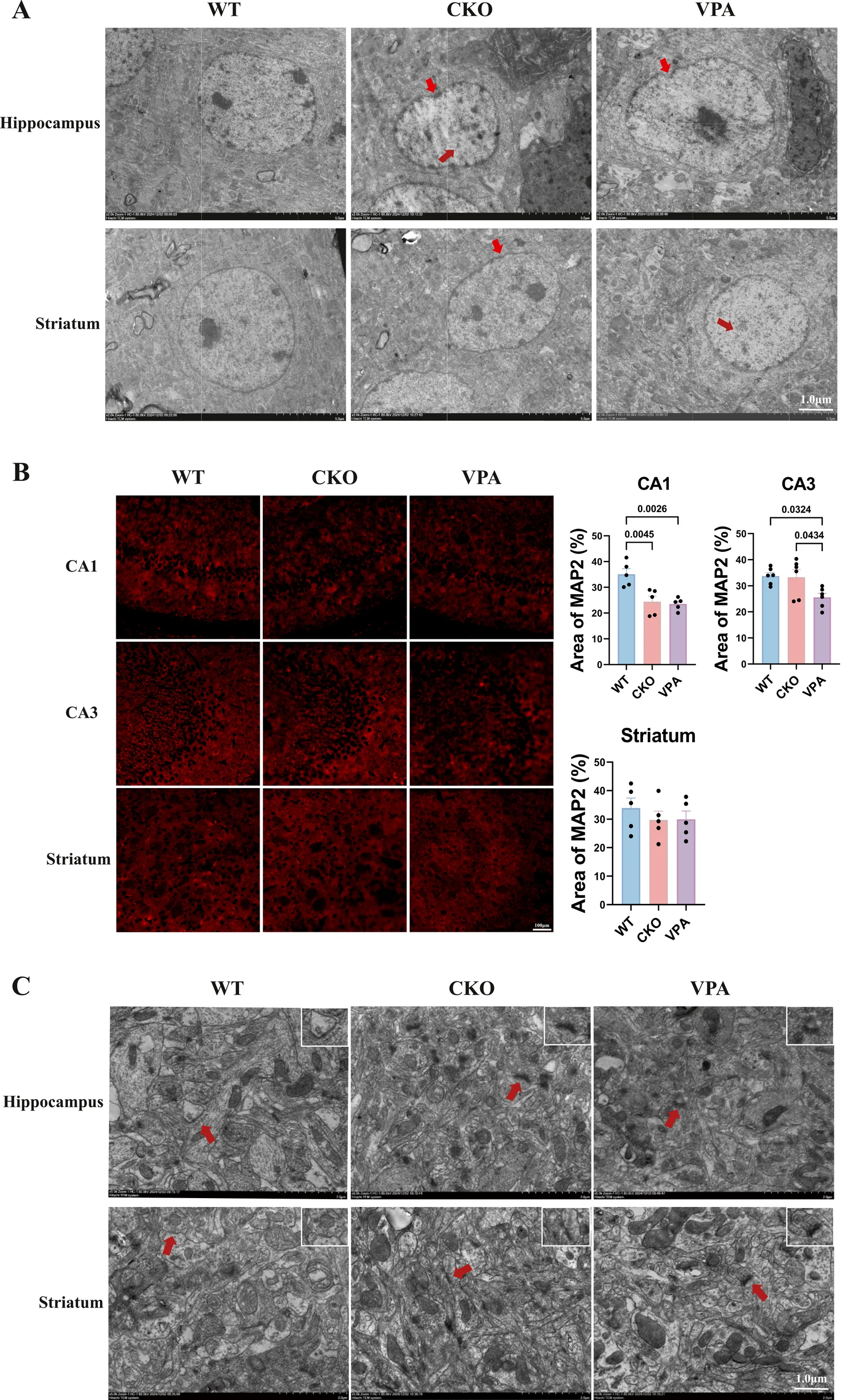
**Neuronal morphological and synaptic ultrastructural alterations in hippocampus and striatum of WT, CKO, and VPA mice.** (A) Transmission electron microscopy images showing neuronal ultrastructure in the hippocampus and striatum (Scale bar, 1.0 μm). (B) Representative immunofluorescence images of MAP2 staining (red) in the hippocampal CA1, CA3, and striatum (Scale bar, 100 μm). Quantification showed significant dendritic atrophy in VPA and CKO mice in the CA1 region; a reduction was also observed in CA3 of VPA mice, but not in CKO mice. No significant changes were found in the striatum. (C) Transmission electron microscopy showing synaptic structures in the hippocampus and stratum. (Scale bar, 1.0 μm). All data are presented as means ± SEM, n = 3 per group (5-6 slices); one-way ANOVA with Turkey post hoc test is used.

To investigate the role of CB1R in modulating GABAergic neurotransmission dynamics, synaptic structure and function, we assessed relevant molecular and ultrastructural markers in the hippocampus and striatum. In hippocampal tissues, protein expressions of VGAT (a GABA transporter) (F_2,12_ = 5.960, *P* = 0.016), GAD67 (GABA synthase) (F_2,12_ = 6.798, *P* = 0.011), and GABARAP (GABA receptor-associated protein) (F_2,12_ = 5.097, *P* = 0.025) in VPA mice were significantly elevated compared to WT mice. CKO mice exhibited a similar trend with significant increase in GAD67 and GABARAP expression, although the elevation in VGAT did not reach statistical significance. Conversely, the levels of ABAT (a key GABA catabolic enzyme) (F_2,12_ = 5.234, *P* = 0.023) were remarkably lower in both VPA and CKO mice relative to WT mice. No significant differences were observed in the levels of GAD65 (another GABA synthase) (F_2,12_ = 0.216, *P* = 0.809) or VGLUT1 (a glutamate transporter protein) (F_2,12_ = 1.110, *P* = 0.361) (Fig. 3A). In the striatum, VGLUT1 (F_2,12_ = 9.228, *P* = 0.004) expression was increased in WT mice compared to VPA mice, but no difference was found between CKO and WT mice. GABARAP (F_2,12_ = 4.921, *P* = 0.028) expression remained unchanged in VPA mice compared to WT mice, whereas was significantly elevated in CKO mice (Fig. 3B). (ABAT, F_2,12_ = 1.710, *P* = 0.222. VGAT, F_2,12_ = 0.024, *P* = 0.976. GAD65, F_2,12_ = 0.898, *P* = 0.433. GAD67, F_2,12_ = 3.387, *P* = 0.068). There was reduced PSD95 (F_2,12_ = 6.666, *P* = 0.011) and elevated Gephyrin (Gep) (F_2,12_ = 11.09, *P* = 0.002) in hippocampus of VPA mice, resulting in a lower PSD95/Gep (F_2,12_ = 10.76, *P* = 0.002) ratio, a pattern recapitulated in CKO mice. While SYN1 (F_2,12_ = 9.896, *P* = 0.003) expression remained unaltered in VPA mice, a significant downregulation was observed in CKO mice (Fig. 3C). In the striatum, PSD95 (F_2,12_ = 4.303, *P* = 0.039) levels and ratio of PSD95/Gep (F_2,12_ = 7.113, *P* = 0.001) were significantly lower in VPA mice than in WT mice, whereas no differences were detected in CKO mice compared to WT (Fig. 3D) (Gep, F_2,12_ = 1.384, *P* = 0.288. SYN1, F_2,12_ = 1.417, *P* = 0.280). We employed GAD67 as a GABAergic neuron marker co-stained with NeuN to examine the distribution and density of mature GABAergic neurons. Both VPA and CKO mice exhibited significantly lower proportion of GAD67^+^NeuN^+^ among NeuN^+^ neurons in the hippocampal CA1 (F_2,21_ = 39.27, *P* < 0.0001) and CA3 (F_2,21_ = 17.69, *P* < 0.0001) regions compared to WT controls (Fig. 4A–B). No significant differences were observed in the striatum (Fig. 4C) (F_2,12_ = 0.022, *P* = 0.978).

**Fig. 5:**
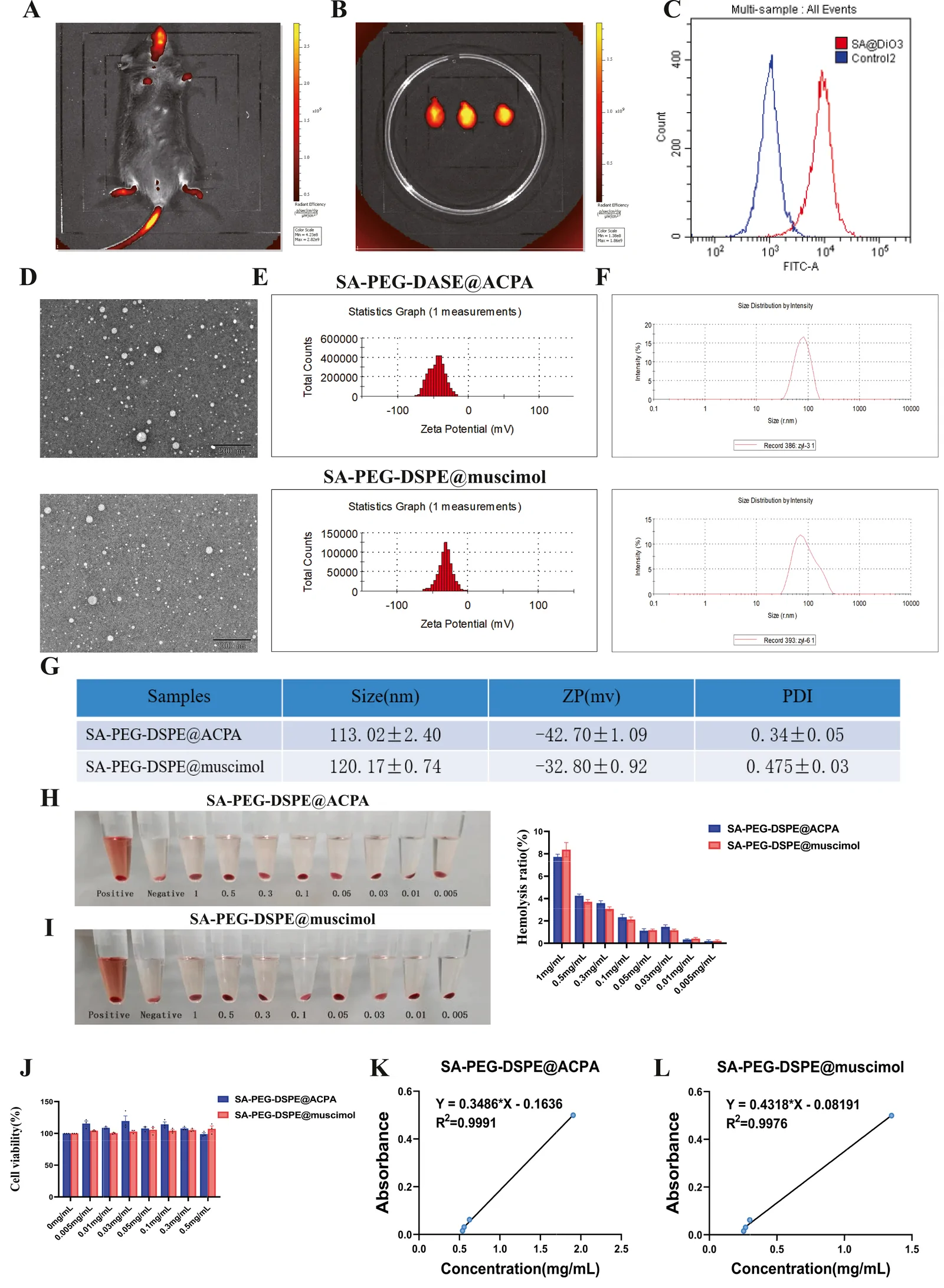
**In vivo and in vitro evaluation and physicochemical characterization of SA-PEG-DSPE@ACPA/muscimmol nanomicelles.** (A and B) In vivo fluorescence imaging confirmed effective brain-targeting of SA-PEG-DSPE@DiR nanomicelles following tail vein injection. (C) Flow cytometry analysis showing uptake capacity of SA-PEG-DSPE@DiO nanomicelles by PC12 cells. (D) Transmission electron microscopy (TEM) image depicting spherical morphology and uniform distribution of SA-PEG-DSPE@ACPA (upper) and SA-PEG-DSPE@muscimol (lower) nanomicelles. (E) Average particle size distribution profile of SA-PEG-DSPE@ACPA/muscimol nanomicelles. (F) Zeta potential measurements of SA-PEG-DSPE@ACPA/muscimol nanomicelles. (G) Summary table showing particle size, polydispersity index (PDI), and zeta potential (ZP). (H and I) Hemolysis assays results indicating minimal hemolytic activity (<5%). Paired student *t*-test. (J) MTT assay demonstrating high cell viability (>90%) and low cytotoxicity of nanomicelles in PC12 cells. (K and L) Standard curves for calculating encapsulation efficiency and drug loading quantification confirming high drug entrapment rates. Unpaired student *t*-test. All data are presented as means ± SEM, n = 3 per group.

Ultrastructural analysis by transmission electron microscopy revealed distinct neuronal structural abnormalities in the hippocampus and striatum. In WT mice, hippocampal neurons exhibited round or oval nuclei with smooth, intact nuclear membranes and uniformly distributed chromatin. In contrast, VPA mice exhibited changes such as invaginated nuclear membranes and clumped chromatin. CKO mice exhibited reduced nuclear volume and uneven chromatin distribution (Fig. 5A). Similar abnormalities were also observed in the striatum of both VPA and CKO mice (Fig. 5A).Dendritic morphology was assessed by MAP2 immunofluorescence. In hippocampal CA1, WT mice displayed a well-organized dendritic architecture with tightly packed spines, whereas VPA mice exhibited marked dendritic atrophy and reduced branching (F_2, 12_ = 11.65, *P* = 0.002). CKO mice showed moderate dendritic atrophy, with a significantly reduced MAP2-positive area similar to VPA mice. In hippocampal CA3, the positive area of VPA mice was significantly lower than WT mice, and there was no statistical difference between CKO and WT mice (Fig. 5B) (F_2, 15_ = 5.045, *P* = 0.021). In the striatum, no significant differences in MAP2 staining were found among groups (Fig. 5B) (F_2, 12_ = 0.538, *P* = 0.597). Electron microscopy of synaptic structures revealed morphological differences in the hippocampus among groups. In WT mice, synapses exhibited well-defined pre- and postsynaptic membranes and uniform postsynaptic density. In contrast, VPA mice displayed thickened postsynaptic density. CKO mice also exhibited thickened postsynaptic density (Fig. 5C). Similar postsynaptic density thickening was observed in the striatum of both VPA and CKO mice (Fig. 5C).

Collectively, the molecular, cellular, and ultrastructural alterations in GABA-CB1^−/−^ mice indicated that conditional knockout of CB1R in GABAergic neurons reproduced key hippocampal features of GABAergic dysfunction relevant to ASD, but not sufficient to account for the full molecular phenotype induced by prenatal VPA exposure.

### Evaluation, characterization, and dosage selection of nanomicelles

**Fig. 6:**
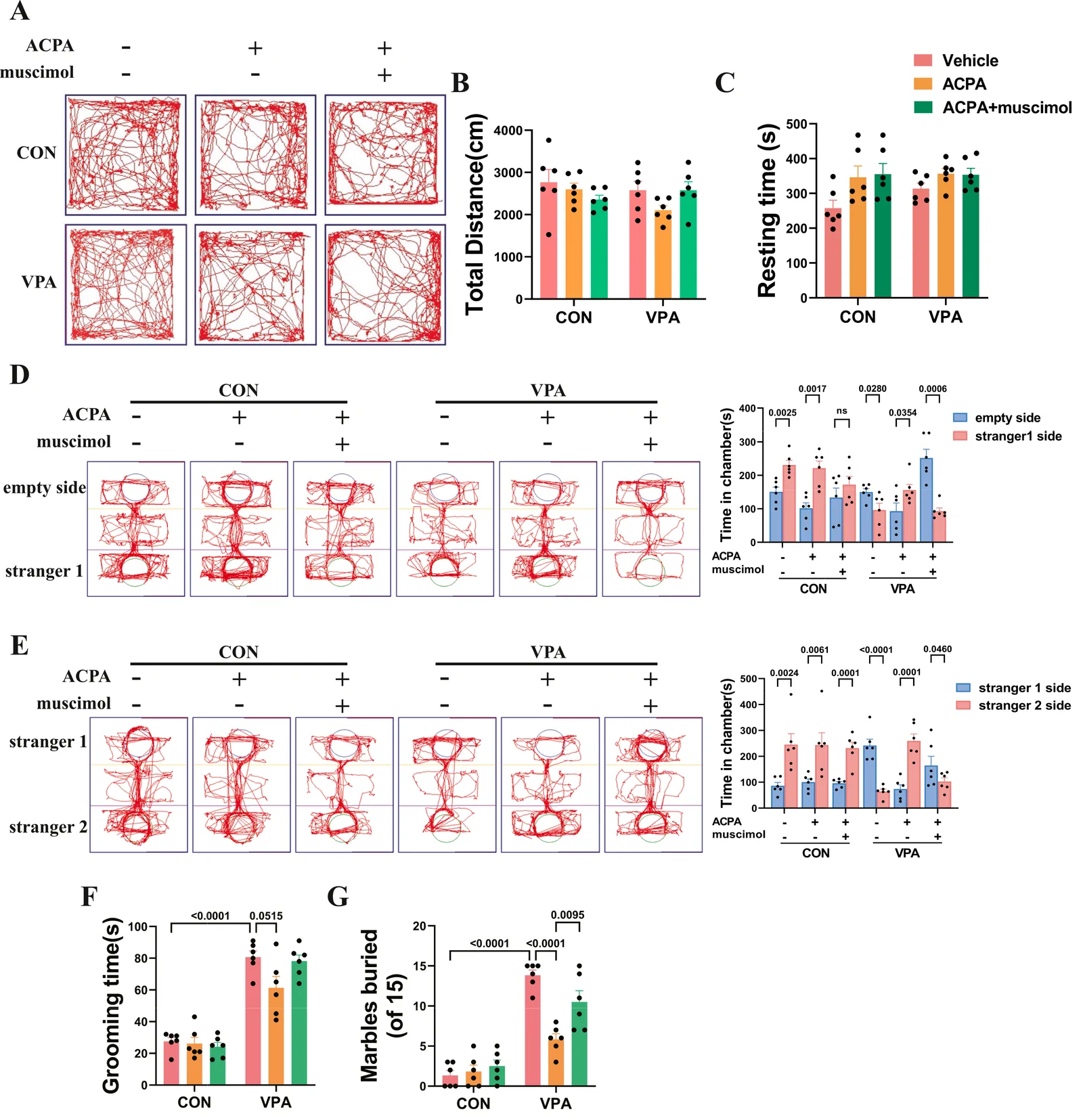
**Effects of CB1R activation and GABAAR stimulation on behavioral deficits in VPA-exposed mice.** (A–B) No significant differences in locomotor activity were observed across groups in the open field test. Shown are representative images (A), resting time (B), and total distance movement (C). Two-way ANOVA. (D) In the sociability test, SA-PEG-DSPE@ACPA treatment significantly increased preference for stranger 1 in VPA mice, while this increase was reduced by co-administration of SA-PEG-DSPE@muscimol. Unpaired student *t*-test. (E) In the social preference test, SA-PEG-DSPE@ACPA-treated VPA mice exhibited increased interaction with stranger 2, however, this trend was attenuated following SA-PEG-DSPE@muscimol co-treatment, though not reaching statistical significance. Unpaired student *t*-test. (F and G) SA-PEG-DSPE@ACPA significantly reduced repetitive behaviors in VPA mice, as indicated by reduced grooming duration (F) and marble-burying (G); these effects were partially attenuated following SA-PEG-DSPE@muscimol co-treatment. Two-way ANOVA. All data are presented as means ± SEM, n = 6 per group; multiple comparisons with Sidak post hoc test.

This study employed SA-PEG-DSPE nanomicelles to deliver ACPA (a CB1R agonist) and muscimol (a GABAAR agonist), aiming to investigate whether the therapeutic effects of CB1R activation on ASD-like behaviors and neuronal damage are mediated via the GABAergic system. In vivo fluorescence imaging with SA-PEG-DSPE@DiR nanomicelles confirmed successful brain targeting following tail vein injection (Fig. 6A–B). In vitro flow cytometry analysis revealed significantly enhanced fluorescence intensity in PC12 cells treated with SA-PEG-DSPE@DiO- compared to controls, indicating efficient cellular uptake (Fig. 6C). The images of TEM showed that both SA-PEG-DSPE@ACPA and SA-PEG-DSPE@muscimol nanomicelles exhibited spherical shape and uniform distribution (Fig. 6D). Particle size, zero potential and polydispersity indices are presented in Fig. 5E–G. Hemolysis and MTT assays demonstrated that the nanomicelles were non-hemolytic (hemolysis rate *<* 5 %) and non-cytotoxic (cell viability *>* 90 %) (Fig. 6H–J). EE (%) was 94.05% for ACPA and 84.62% for muscimol, and LE (%) was 12.27% and 10.91%, respectively (Fig. 6K-L), which indicating high encapsulation capacity and stability. These results supported biosafety and brain-targeting capacity of the SA-PEG-DSPE nanomicelle delivery system.

### CB1R activation ameliorated ASD-like behaviors and neuronal deficits accompanied by modulation of GABA signaling

**Fig. 7:**
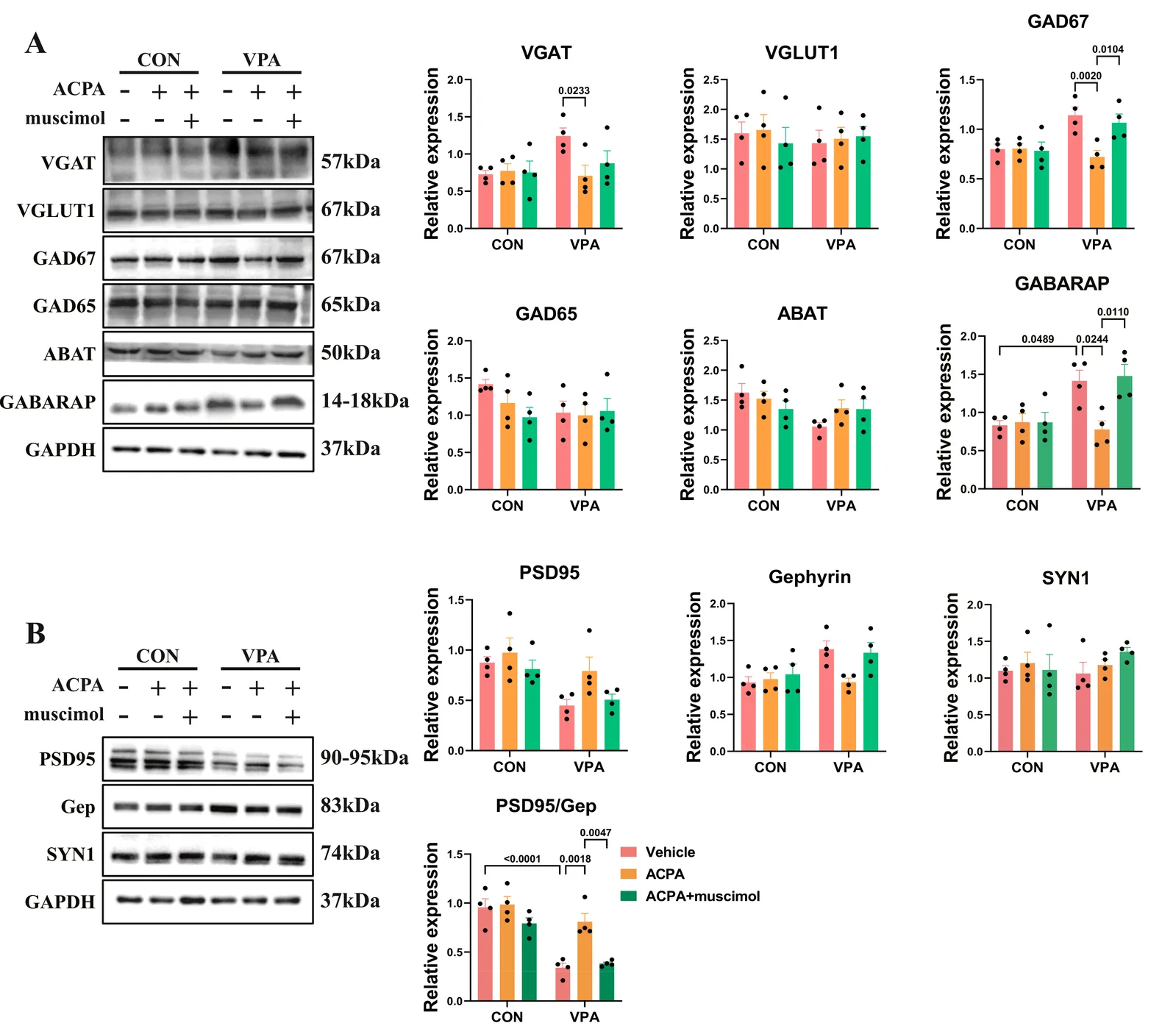
**Effects of CB1R activation and GABAAR stimulation on GABAergic and synaptic protein expression in VPA-exposed mice.** (A) Representative immunoblots and quantification of GABAergic-related proteins in the hippocampus. SA-PEG-DSPE@ACPA treatment significantly reduced the elevated expression of GAD67, VGAT and GABARAP in VPA mic. Following co-treatment with SA-PEG-DSPE@muscimol, the reductions in GAD67 and GABARAP were less pronounced. Two-way ANOVA. (B) Representative immunoblots and quantification of synaptic proteins in the hippocampus. SA-PEG-DSPE@ACPA reduced Gep expression and significantly increased the PSD95/Gep ratio in VPA mice, indicative of synaptic restoration. Following co-administration of SA-PEG-DSPE@muscimol, these changes were less pronounced. Two-way ANOVA. All data are presented as mean ± SEM, n = 4 per group; multiple comparisons with Sidak post hoc test.

The SA-PEG-DSPE@ACPA/muscimol nanomicelles intervention did not elicit significant ASD-like behavioral and biological changes in control mice. In the open field test, no significant changes in locomotor activity were found across all groups before or after treatment (Fig. 7A–C) (Total Distance, F_Interaction__(2, 30)_ = 1.751, *P* = 0.191, F_Genotype (1, 30)_ = 0.980, *P* = 0.330, F_Treatment (2, 30)_ = 1.422, *P* = 0.257; Resting time, F_Interaction__(2, 30)_ = 0.830, *P* = 0.446, F_Genotype (1, 30)_ = 1.235, *P* = 0.275, F_Treatment (2, 30)_ = 5.336, *P* = 0.010). In the three-chamber test, SA-PEG-DSPE@ACPA administration significantly corrected social deficits in VPA mice, as evidenced by increased time spent in the mouse-containing chamber during the sociability test and restored preference for novel over familiar conspecifics during the social preference test. While, co-administration of SA-PEG-DSPE@muscimol partially attenuated these pro-social effects of CB1R activation (Fig. 7D–E). Regarding stereotyped behaviors, two-way ANOVA indicated that a main effect of group (VPA vs. CON), with VPA mice displaying more repetitive behaviors. VPA mice treated with SA-PEG-DSPE@ACPA exhibited significantly reduced grooming duration (F_Interaction__(2, 30)_ = 3.040, *P* = 0.063, F_Genotype (1, 30)_ = 181.3, *P* < 0.0001, F_Treatment (2, 30)_ = 3.048, *P* = 0.062) and fewer marbles buried (F_Interaction__(2, 30)_ = 12.07, *P* = 0.0001, F_Genotype (1, 30)_ = 133.6, *P* < 0.0001, F_Treatment (2, 30)_ = 9.944, *P* = 0.0005), suggesting a mitigation in repetitive behaviors. This improvement was partially reserved by co-treatment with SA-PEG-DSPE@muscimol (Fig. 7F–G). Altogether, these results suggested that CB1R activation ameliorated core ASD-like behavioral deficits, namely social impairments, and stereotyped behaviors, while simultaneous activation GABAAR might counteract these therapeutic effects.

**Fig. 8:**
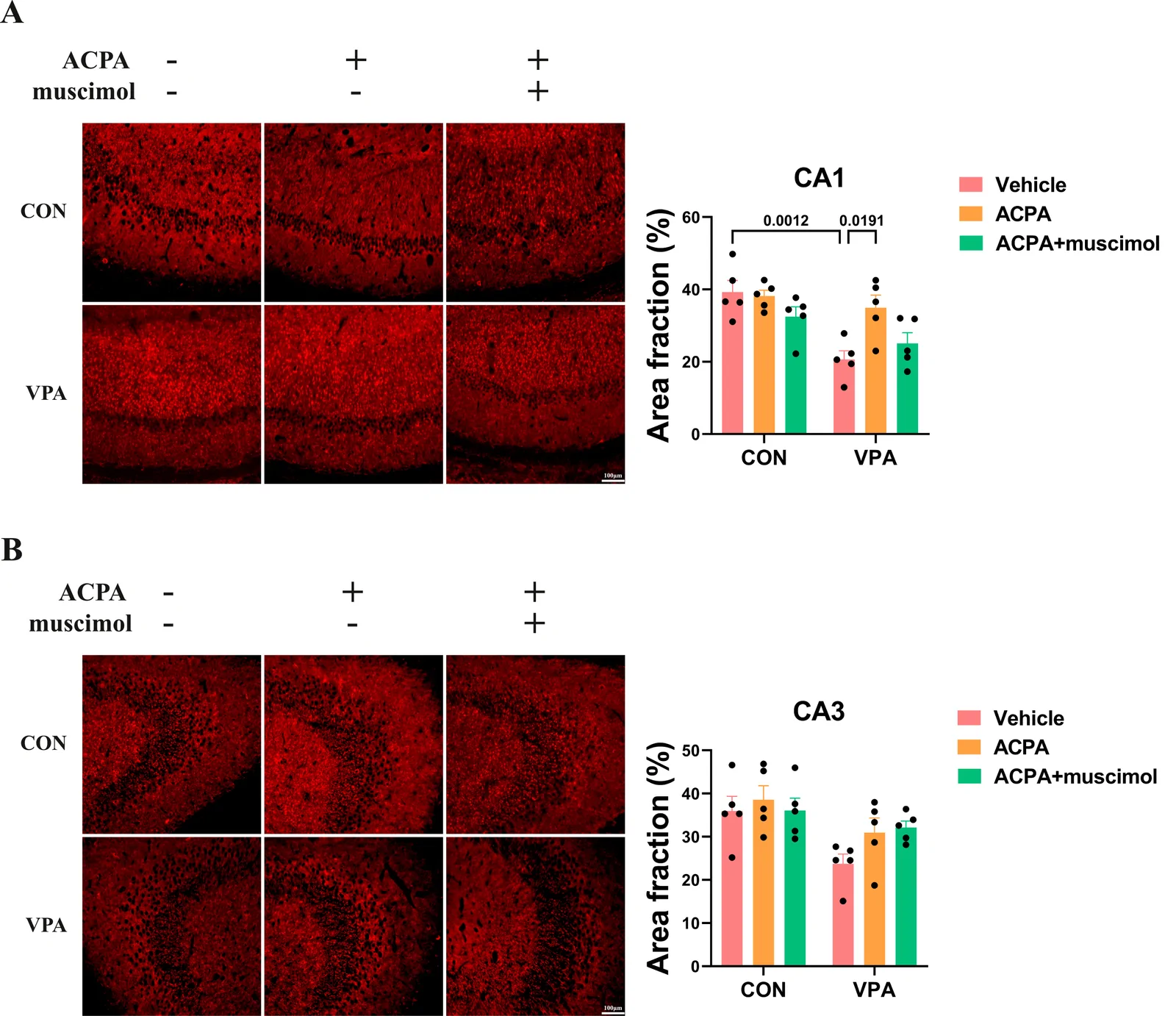
**CB1R activation restored dendritic integrity in the hippocampal CA1 region of VPA mice.** (A) Representative immunofluorescence images showing MAP2 staining (red) in the hippocampal CA1 region (Scale bar, 100 μm). Treatment of SA-PEG-DSPE@ACPA significantly increased MAP2-positive area in VPA mice. Co-administration of SA-PEG-DSPE@muscimol decreased this effect, although the difference did not reach statistical significance. Two-way ANOVA. (B) Representative immunofluorescence images showing MAP2 staining (red) in hippocampal CA3 region. (Scale bar, 100 μm). SA-PEG-DSPE@ACPA partially reversed dendritic atrophy in VPA mice, while co-treatment with SA-PEG-DSPE@muscimol reduced this benefit without statistical significance. Two-way ANOVA. All data are presented as mean ± SEM, n = 3 per group, 1-2 slices; multiple comparisons with Sidak post hoc test.

There was a significant main effect of group on protein expression, which showed significantly less of the ABAT (F_Interaction (2, 18)_ = 2.345 *P* = 0.124, F_Genotype (1, 18)_ = 4.870, *P* = 0.041, F_Treatment (2, 18)_ = 0.353 *P* = 0.707) and PSD95/Gep (F_Interaction (2, 18)_ = 5.225 *P* = 0.016, F_Genotype (1, 18)_ = 52.18 *P* < 0.0001, F_Treatment (2, 18)_ = 11.69, *P* = 0.0006) ratio and the increase of GAD67 (F_Interaction (2, 18)_ = 5.068 *P* = 0.018, F_Genotype (1, 18)_ = 9.215 *P* = 0.007, F_Treatment (2, 18)_ = 4.492, *P* = 0.026), GABARAP (F_Interaction (2, 18)_ = 5.334 *P* = 0.015, F_Genotype (1, 18)_ = 13.42 *P* = 0.002, F_Treatment (2, 18)_ = 4.791 *P* = 0.022), PSD95 (F_Interaction (2, 18)_ = 0.763 *P* = 0.481, F_Genotype (1, 18)_ = 14.29 *P* = 0.001, F_Treatment (2, 18)_ = 3.377, *P* = 0.057) and Gep (F_Interaction (2, 18)_ = 2.828 *P* = 0.086, F_Genotype (1, 18)_ = 7.329 *P* = 0.014, F_Treatment (2, 18)_ = 2.827, *P* = 0.086) in VPA mice. Moreover, SA-PEG-DSPE@ACPA treatment significantly reduced the elevated protein expression of VGAT (F_Interaction (2, 18)_ = 2.756 *P* = 0.090, F_Genotype (1, 18)_ = 3.410, *P* = 0.081, F_Treatment (2, 18)_ = 1.961, *P* = 0.170), GAD67, and GABARAP in VPA mice. Co-administration of SA-PEG-DSPE@muscimol reversed the suppression of GAD67 and GABARAP, while VGAT expression remained unaffected (Fig. 8A). In addition, SA-PEG-DSPE@ACPA enhanced the PSD95/Gep ratio, indicating CB1R activation effectively mitigated excitatory/inhibitory synaptic imbalance in the molecular level. This effect was attenuated by co-administration SA-PEG-DSPE@muscimol, which contributed to a decrease in the PSD95/GEP ratio (Fig. 8B) (GAD65, F_Interaction (2, 18)_ = 1.350 *P* = 0.284, F_Genotype (1, 18)_ = 1.817 *P* = 0.194, F_Treatment (2, 18)_ = 1.130 *P* = 0.345. VGLUT1, F_Interaction (2, 18)_ = 0.267 *P* = 0.769, F_Genotype (1, 18)_ = 0.127 *P* = 0.726. F_Treatment (2, 18)_ = 0.090 *P* = 0.914. SYN1, F_Interaction (2, 18)_ = 0.772 *P* = 0.477, F_Genotype (1, 18)_ = 0.337 *P* = 0.569, F_Treatment (2, 18)_ = 0.728 *P* = 0.496). Two-way ANOVA showed significant main effect of group, while within vehicle condition, VPA mice showed significantly lower of MAP2-positive area in CA1 compared with CON mice. Immunofluorescence analysis further revealed that SA-PEG-DSPE@ACPA significantly restored dendritic structure in the CA1 region (Interaction, F_Interaction (2, 24)_ = 4.129, *P* = 0.029. F_Genotype (1, 24)_ = 18.49, *P* = 0.0002. F_Treatment (2, 24)_ = 4.548, *P* = 0.021) of VPA mice, as indicated by an increased MAP2-positive area, reflecting enhanced dendritic complexity. Meanwhile, co-treatment with SA-PEG-DSPE@muscimol tended to diminish this effect, the difference did not reach statistical significance (Fig. 9A–B) (F_Interaction (2, 24)_ = 1.058, *P* = 0.363. F_Genotype (1, 24)_ = 11.46, *P* = 0.002. F_Treatment (2, 24)_ = 1.720, *P* = 0.201). The above results showed that CB1R activation ameliorated dendritic structural abnormalities and synaptic dysfunction in ASD model mice, whereas GABAAR activation may counteract these neuroprotective effects.

## Discussion

This study demonstrated that conditional knockout of CB1R in forebrain GABAergic neurons resulted in core ASD-like behavioral phenotypes analogous to those observed in VPA-induced models, including impaired social interaction, repetitive behaviors, and cognitive deficits. These phenotypes coincided with disruptions in GABAergic transmission, synaptic integrity, and neuronal ultrastructure, predominantly, in the hippocampus but absent in striatal areas, suggesting that the hippocampus may represent a region particularly affected by CB1R-associated GABAergic alterations. These findings suggested that an important contribution of GABAergic CB1R deficiency to ASD-like pathophysiology. Subsequently, utilizing a brain-targeted nanomaterial delivery system, we further demonstrated that ACPA administration effectively improved GABAergic signaling, dendritic architecture, and ASD-like behavioral deficits in VPA-induced models, whereas co-administration of GABAAR agonist muscimol partially attenuated these therapeutic benefits. These findings suggested that nanomicelle-mediated CB1R activation ameliorated VPA-induced behavioral and neuronal deficits, with GABAergic signaling potentially contributing to these effects.

Based on our previous findings, hypofunction of the endocannabinoid system (eCB) has been consistently observed in both human ASD patients and corresponding animal models (VPA-exposed offspring and BTBR T + ltpr3tf/J mouse). This hypofunction, mainly characterized by abnormal CB1R signaling, was associated with autistic features [22,23]. Furthermore, conventional CB1R knockout mice exhibited notable ASD core behavioral abnormalities and cognitive deficits [19,24,25]. More specifically, Albayram et al. reported conditional deletion of CB1R in GABAergic neurons resulted in impaired spatial learning and reduced memory flexibility in the Morris water maze, as well as deficits in social partner recognition in the novel object recognition experiment [26]. Another study demonstrated that hippocampal GABAergic CB1R was involved in associative learning processes [27]. Similarly, Cuadrado and colleagues demonstrated loss of CB1R in inhibitory neurons disrupted hippocampal-dependent cognition while locomotor activity in open-field tests remained unaffected [28]. In addition, other groups also reported that conditional deletion of CB1R in GABAergic neurons elicited social abnormalities [29]. Our findings were consistent with these reports, as GABA-CB1^−/−^ mice in our study exhibited robust social interaction deficits and impaired spatial learning and memory, paralleling the behavioral impairments observed in VPA-induced ASD model mice. Importantly, we extended these previous observations by demonstrating that GABA-CB1^−/−^ mice also displayed pronounced repetitive and stereotyped behaviors. This novel finding highlighted a broader spectrum of ASD-like phenotypes associated with CB1R deficiency in GABAergic neurons.

At the molecular level, both VPA and GABA-CB1^−/−^ mice showed upregulation of GAD67 and GABARAP, coupled with downregulation of ABAT. These coordinated shifts in key regulatory components suggest a net elevation in local GABA levels, consistent with earlier observations in other ASD models, such as maternal immune activation (MIA) [6,30]. In addition, the decreased PSD95/Gep ratio and reduced GAD67^+^ neuronal density, along with abnormal neuronal and synaptic structure, observed in our study are consistent with molecular evidence of E/I imbalance, a feature frequently associated with hippocampal dysfunction in ASD [15,17,18]. Our findings in GABA-CB1^−/−^ mice suggest a potential involvement of CB1R in the regulation of GABAergic homeostasis. The decreased E/I balance we observed in these mice aligned with previous reports from other CB1R-deficient models, in which increased frequency of spontaneous inhibitory postsynaptic currents was detected in CA1 pyramidal neurons [26,[31], [32], [33]]. Developmentally, CB1R expression on the axons and growth cones of cortical GABAergic interneurons has been shown to facilitate tangential and radial migration during late embryonic development; while loss of CB1R signaling resulted in defective postsynaptic target selection [34]. Consistent with these findings, we observed a reduction in GAD67^+^ neurons, disruption of dendritic architecture, as well as abnormal neuronal and synaptic morphology in GABA-CB1^−/−^ mice, which further supports an association between CB1R deficiency and disrupted GABAergic neuronal development. Collectively, these findings suggest a functional interplay between CB1R and GABAergic signaling, although they do not establish a direct linear causal relationship.

In GABA-CB1^−/−^ mice, we identified a spectrum of alterations comparable to those observed in VPA-induced model mice, however these changes were confined specifically to the hippocampus, with no significant effects detected in the striatum. This regional discrepancy may be explained by differential CB1R expression patterns. First, the cell-type distribution of CB1R differs markedly across brain regions. In the hippocampus, GABAergic interneurons, particularly cholecystokinin positive (CCK^+^) subtype, are predominant, where CB1R are abundantly expressed on [35]. In contrast, striatal interneurons are dominated by parvalbumin positive [PV^+^] or somatostatin positive [SOM^+^] subtypes, which express little or lack CB1R [36]. Accordingly, the functional mechanisms maintaining inhibitory balance differ between the hippocampus and striatum. In the hippocampus, GABAergic regulation strongly depends on CB1R signaling in CCK^+^ interneurons, which shapes inhibitory transmission, network oscillations, and synaptic plasticity [35,37]. Thus, loss of CB1R directly disrupts hippocampal inhibitory homeostasis and microcircuit integrity, leading to ASD-like phenotypes. Although CB1R is present in the striatum overall and the eCB signaling contributes significantly to striatal plasticity, CB1R is mainly localized to presynaptic terminals of dopaminergic medium spiny neurons (MSNs) and glutamatergic neurons, with CB1Rs in this region playing a leading role in diminution in excitatory transmission [36,[38], [39], [40]]. Moreover, the core striatal circuitry relies predominantly on strong inhibition from PV^+^ fast-spiking interneurons onto MSNs, a pathway that operates largely independent of CB1R modulation [41]. As a result, even in the absence of CB1R, the fundamental GABAergic functions of the striatum can be maintained. Abovementioned findings may explain why CB1R deficiency was associated with more pronounced hippocampal GABAergic dysfunction and related social and cognitive behaviors, while leaving striatal GABAergic activity relatively preserved.

Given the correlative evidence linking CB1R deficiency to altered GABAergic features, we next explored whether pharmacological CB1R activation was associated with improvement in these abnormalities. With the aim of facilitating central pharmacological modulation and potentially reducing peripheral confounding effects associated with systemic administration, both ACPA and muscimol were delivered using brain-targeted SA-PEG-DSPE nanomicelles. Although our results supported the brain-accessible capability of the nanocarrier, the brain distribution of the encapsulated drugs was not directly determined, preferential central drug exposure and reduced peripheral exposure cannot be established from the present data. In VPA-exposed offspring, nanomicelle-mediated ACPA administration was associated with partial normalization of GABAergic molecular alterations and dendritic abnormalities, and alongside improvements in sociability and reduced stereotypy. These outcomes were consistent with prior evidence showing that CB1R activation mitigated GABAergic dysfunction and behavioral abnormalities. For example, Saez et al. reported that prenatal exposure to the CB1R agonist WIN55,212-2 elevated GABAergic neuron density [42], while in a Huntington’s disease model, WIN55,212-2 treatment ameliorated hippocampal dendritic spine pathology, restored PSD95 expression, and reduced depolarization-induced suppression of inhibition [43]. Similarly, our prior findings have shown that broad CB1R activation rescued social deficits and cognitive impairments in rodent ASD models [19,44]. Together, these findings provide supportive evidence that pharmacological CB1R activation is associated with alleviation of GABAergic molecular deficits, and ASD-like behavioral phenotypes in VPA-exposed offspring, suggesting CB1R-GABAergic interaction may represent a potential therapeutic target worthy of further investigation in ASD.

To further explore the potential functional interplay between CB1R and GABAergic signaling, we co-treated with CB1R agonist ACPA and GABAAR agonist muscimol to VPA-exposed offspring. GABAA receptors serve as the major inhibitory neurotransmitter receptors in the brain, maintaining inhibitory tone and neuronal network homeostasis [45,46]. Dysregulated GABAAR signaling has been implicated in ASD-related pathological processes [47,48], a phenomenon notably observed in both GABA-CB1^−/−^ mice and the VPA-induced model in this study. Nanomicelle-mediated pharmacological treatment with ACPA was associated with reduced GABAAR expression in VPA-exposed offspring, while muscimol co-administration appeared to enhance GABAAR-mediated inhibitory signaling, and partially attenuate the behavioral improvements by CB1R activation. Yu and colleagues reported that the functional relevance of the CB1R-GABAAR-dependent pathway may account for the regulation of hippocampal synaptic plasticity, and cognition and memory [49]. Additionally, muscimol has been reported to ameliorate behavioral abnormalities in certain neurodevelopmental disorder models, suggesting the partial loss of ACPA-mediated rescue observed in the present study is unlikely to reflect nonspecific behavioral deterioration caused by muscimol alone [50]. Rather, these findings suggest that the beneficial effects of CB1R activation may depend on finely regulated inhibitory signaling states, rather than generalized enhancement of inhibition. Together, these observations are consistent with a potential involvement of GABAAR-related signaling in the functional changes associated with CB1R modulation of ASD-like phenotypes. Our findings further suggested that CB1R may participate in the regulation of GABAergic function in ASD, whereas broad pharmacological activation of GABAARs may alter this delicate balance of inhibitory signaling.

In contrast to the broad and persistent ASD-like deficits observed in the VPA-induced model, the phenotypic abnormalities in GABA-CB1^−/−^ mice appeared more restricted. VPA exposure is thought to disrupt neurodevelopment via widespread epigenetic alterations at critical embryonic stage [[51], [52], [53]]. This acute, non-specific insult likely altered neurodevelopmental trajectories, leading to widespread circuit abnormalities that manifested as severe and pervasive behavioral deficits, synaptic dysfunction, and ultrastructural pathology [54,55]. By contrast, the deficits in GABA-CB1^−/−^ mice were primarily induced by cell-specific genetic deletion, confined primarily to CB1R-dependent GABAergic signaling. As the loss of CB1R was present throughout embryogenesis, compensatory adaptation in alternative receptors or pathways may partially recalibrate inhibitory circuit function. Thus, while both models converged on disrupted GABAergic signaling, this developmental plasticity may partly explain why the GABA-CB1^−/−^ phenotype, remained comparatively restricted in scope and severity.

Our study presented several limitations requiring consideration. First, nanomicelle-based delivery of pharmacological agonists may not fully mimic eCB dynamics and could introduce off-target effects, while the lack of direct pharmacokinetic measurements precludes confirmation of brain-selective drug exposure or exclusion of peripheral effects. Second, although our findings support an association between CB1R modulation and GABAergic alterations, we did not perform region- or cell type-specific manipulation of CB1R or directly determine whether GABAAR blockade prevented the behavioral effects of ACPA. The proposed CB1R-GABAAR interplay requires further validation. Third, our assessment of E/I balance relied primarily on protein expression data and lacked direct functional validation, such as electrophysiological recordings of synaptic currents. Finally, GABAergic neurons were examined at a general level without distinguishing subtype-specific interneurons (e.g., PV+, SST+, or CCK+), which may differ in CB1R expression and regulatory profiles. Future studies incorporating electrophysiological analyses with hippocampus-specific and cell type-specific genetic or pharmacological manipulations of CB1R and GABAAR signaling will be essential to determine the causal contribution of GABAergic CB1R-GABAAR signaling to ASD-related phenotypes.

## Conclusion

Using complementary genetic and pharmacological approaches, the present study povides convergent evidence that CB1R is involved in ASD-like behavioral deficits and related neural impairments, with more pronounced GABAergic dysfunction observed in the hippocampus. Nanomicelle-mediated CB1R activation ameliorated GABAergic-related molecular abnormalities and concurrent improvements in behavioral and molecular deficits, while, broad activation of GABAA receptors partially attenuated these beneficial effects. These results suggest that GABAAR-related signaling may participate in the functional association between CB1R modulation and GABAergic alterations in ASD-relevant pathophysiology. Consequently, this study supports further investigation of CB1R as a potential therapeutic target for ASD, and provides a foundation for future studies using cell type- and region-specific genetic or pharmacological approaches to clarify the precise mechanisms underlying CB1R-GABAergic interactions.

## Availability of data and materials

The data analyzed during the current study are available from the corresponding author on reasonable request.

## CRediT author statement

**Jingyi Hu**: Writing – original draft, Validation, Methodology. **Haoran Wang:** Methodology, Data curation. **Yi Jiang**: Formal analysis. **Tianyu Liu**: Formal analysis. **Junyu Ren**: Validation. **Yilin Zhang**: Formal analysis. **Yuting Zhang**: Formal analysis. **Han Zhang**: Formal analysis. **Caihong Sun**: Supervision, Funding acquisition, Conceptualization. **Mingyang Zou**: Writing – review & editing, Funding acquisition, Conceptualization.

## Consent for publication

Not applicable.

## Ethics approval and consent to participate

All animal experiments were conducted in compliance with the Institutional Review Board of Harbin Medical University for Medical Sciences (HMUIRB20200007).

## Funding

This work was supported by Natural Science Foundation of Heilongjiang Province (Grant No. LH2023H017, PL2026H291).

## Declaration of competing interest

The authors declare that they have no competing interests.
