# Supplementary material for: Genetic and pharmacological evidence linking CB1R signaling to hippocampal GABAergic dysfunction in ASD mouse model
Source: Neurotherapeutics. 2026 Sep 18;23(6):e01067. doi: 10.1016/j.neurot.2026.e01067 (PMC13602063; doi:10.1016/j.neurot.2026.e01067)
Supplement: Supplementary file 1 [file mmc2.pdf]

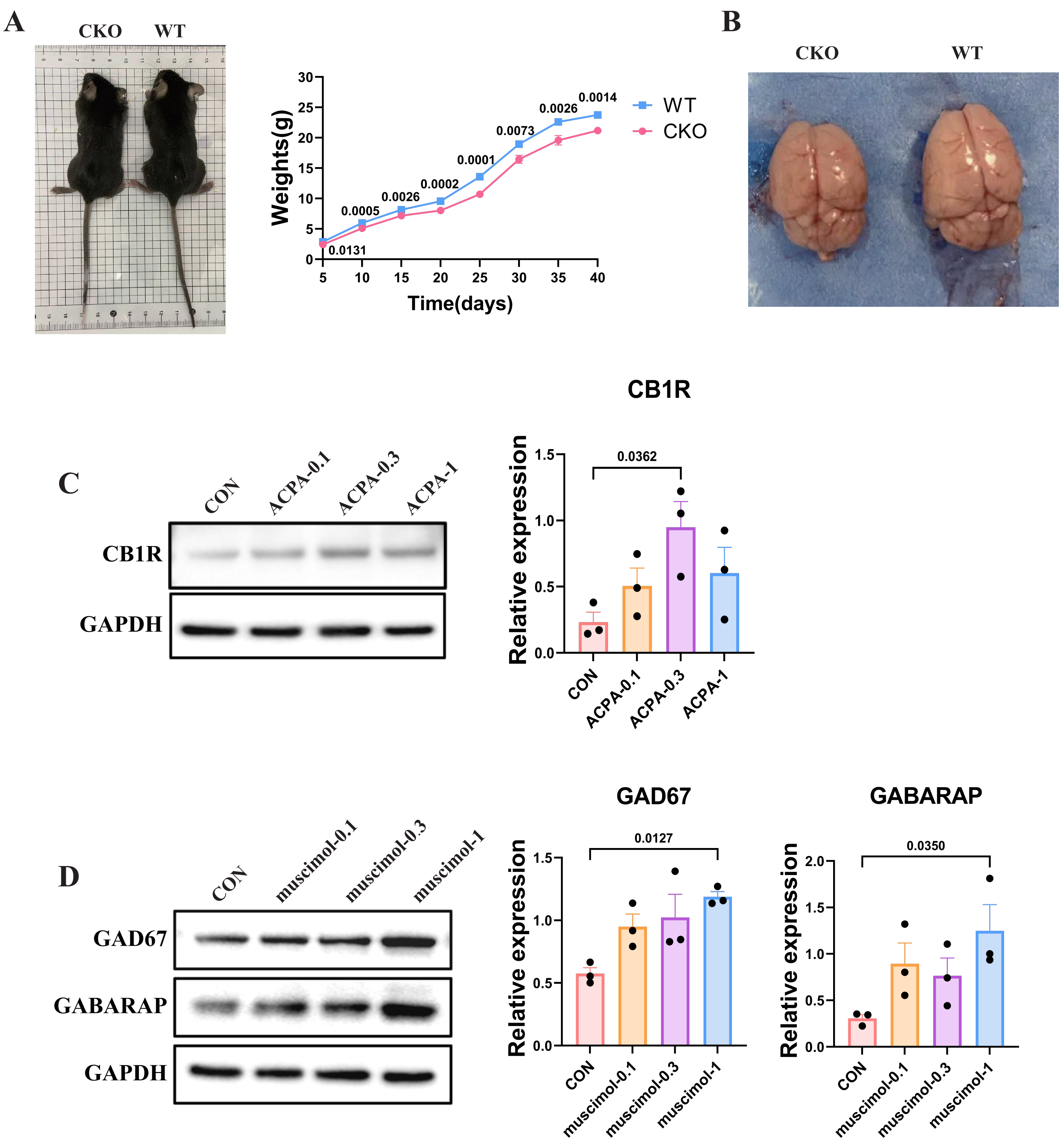
A
CKO
WT
Weights(g)
Time(days)
WT
CKO
0.0131
0.0005
0.0026
0.0002
0.0001
0.0073
0.0026
0.0014
B
CKO
WT
C
CON
ACPA-0.1
ACPA-0.3
ACPA-1
CB1R
GAPDH
CB1R
Relative expression
0.0362
D
CON
muscimol-0.1
muscimol-0.3
muscimol-1
GAD67
GABARAP
GAPDH
GAD67
Relative expression
0.0127
GABARAP
Relative expression
0.0350
