# Supplementary material for: Genetic and pharmacological evidence linking CB1R signaling to hippocampal GABAergic dysfunction in ASD mouse model
Source: Neurotherapeutics. 2026 Sep 18;23(6):e01067. doi: 10.1016/j.neurot.2026.e01067 (PMC13602063; doi:10.1016/j.neurot.2026.e01067)
Supplement: Supplementary file 2 [file mmc3.pdf]

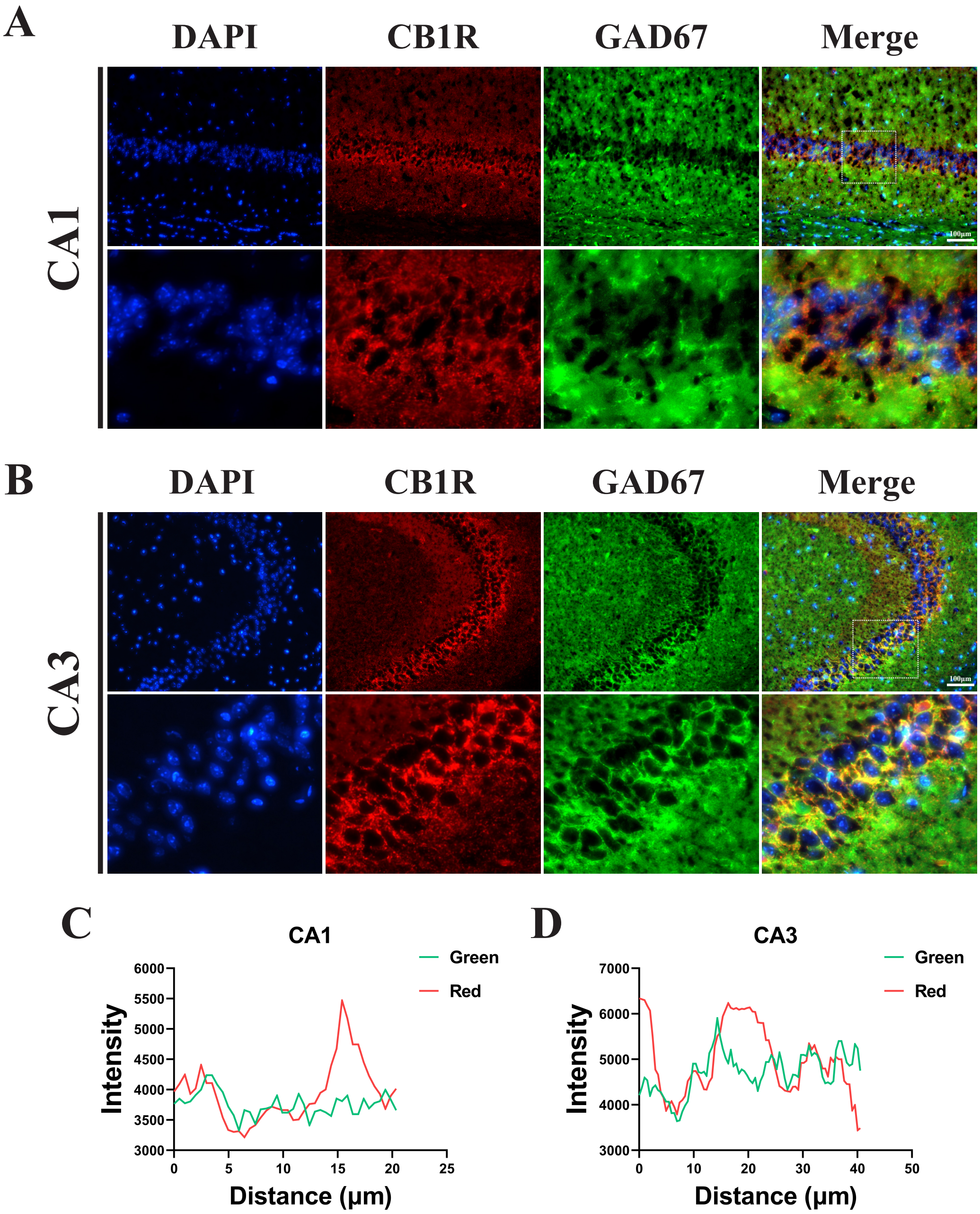
A
DAPI
CB1R
GAD67
Merge
CA1
B
DAPI
CB1R
GAD67
Merge
CA3
C
CA1
Green
Red
Intensity
Distance (μm)
D
CA3
Green
Red
Intensity
Distance (μm)
