# Supplementary material for: Genetic and pharmacological evidence linking CB1R signaling to hippocampal GABAergic dysfunction in ASD mouse model
Source: Neurotherapeutics. 2026 Sep 18;23(6):e01067. doi: 10.1016/j.neurot.2026.e01067 (PMC13602063; doi:10.1016/j.neurot.2026.e01067)

Three -chamber Test

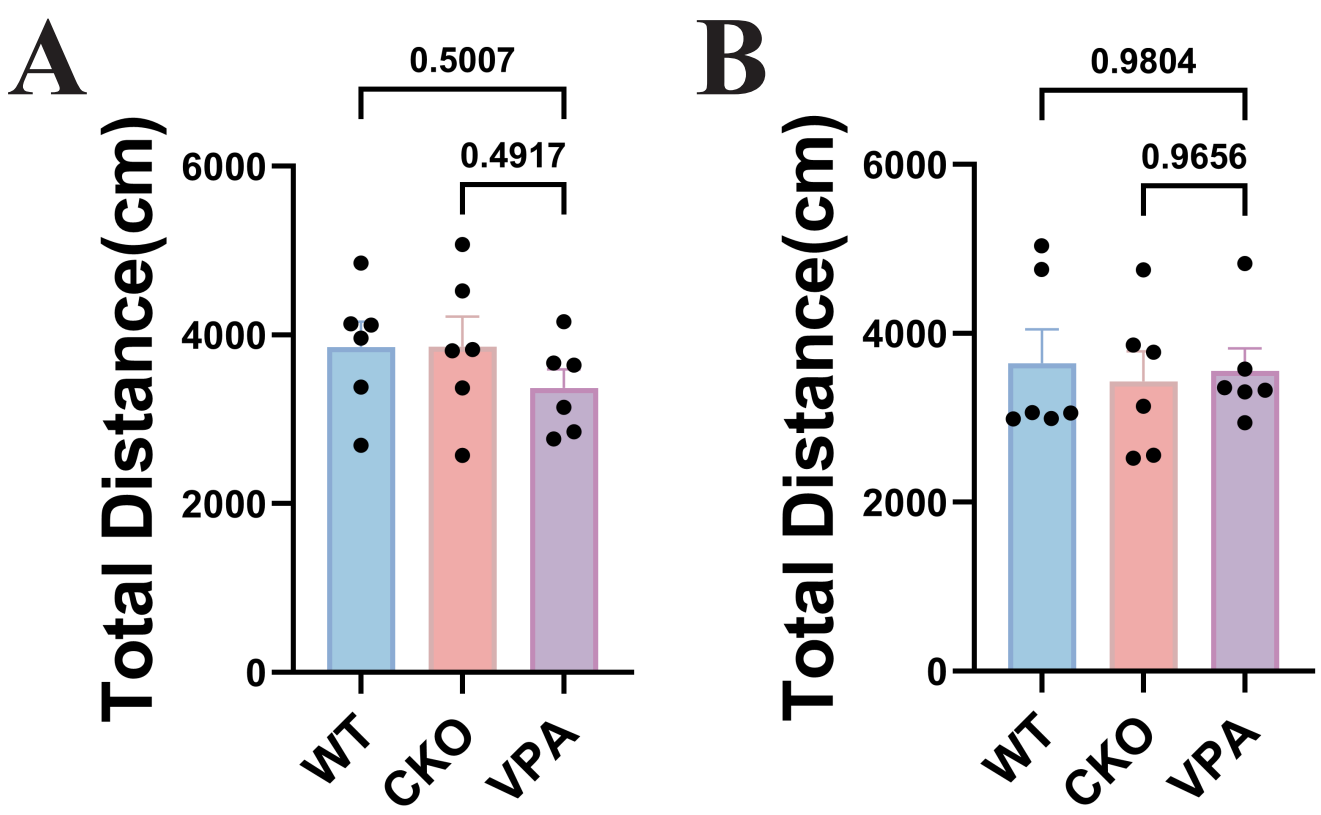

Supplement: Supplementary file 3 [file mmc4.pdf]
