# Supplementary material for: Genetic and pharmacological evidence linking CB1R signaling to hippocampal GABAergic dysfunction in ASD mouse model
Source: Neurotherapeutics. 2026 Sep 18;23(6):e01067. doi: 10.1016/j.neurot.2026.e01067 (PMC13602063; doi:10.1016/j.neurot.2026.e01067)
Supplement: Supplementary file 4 [file mmc5.pdf]

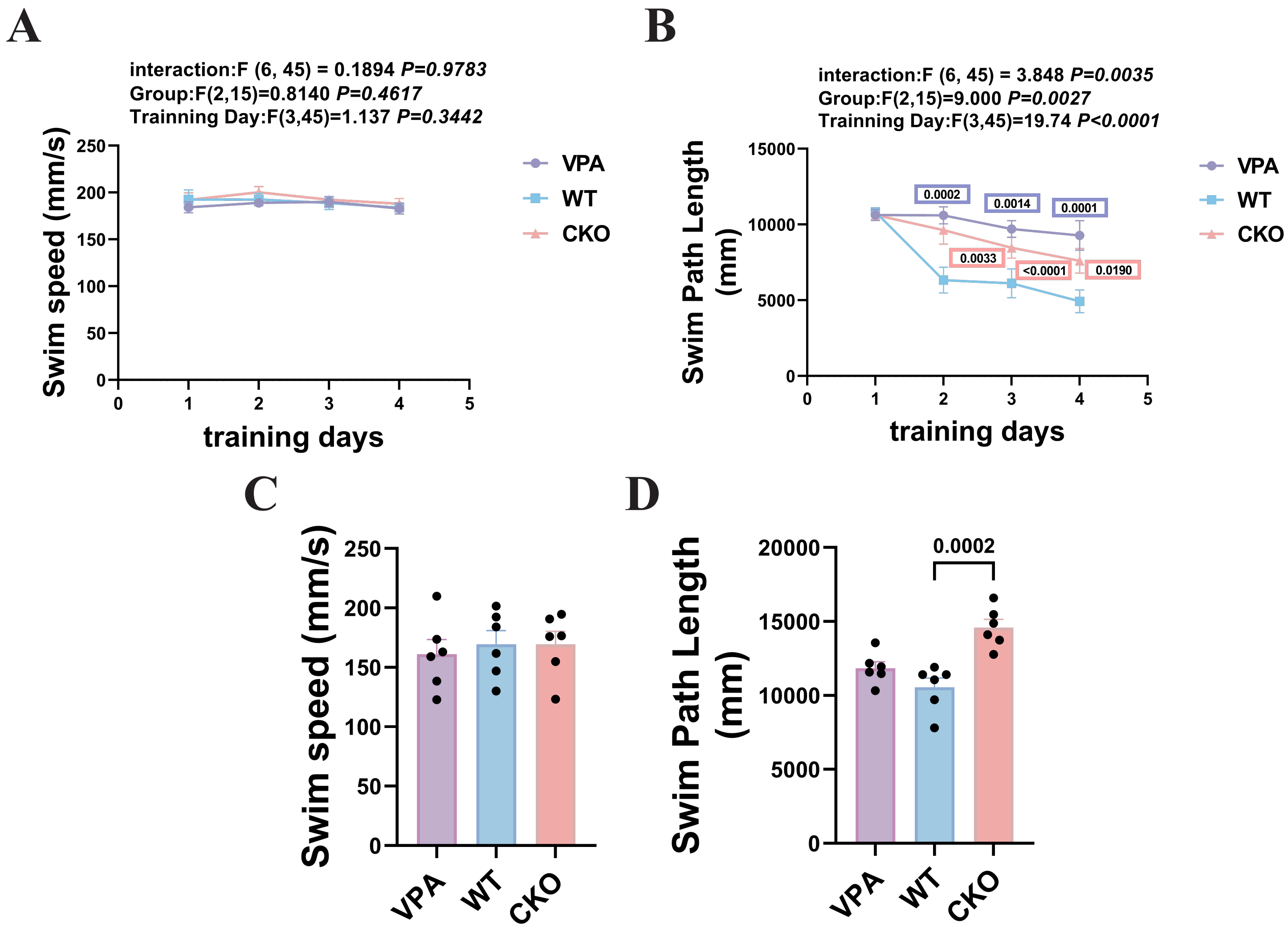

A
interaction:F (6, 45) = 0.1894 P=0.9783
Group:F(2,15)=0.8140 P=0.4617
Trainning Day:F(3,45)=1.137 P=0.3442
Swim speed (mm/s)
training days
VPA
WT
CKO
B
interaction:F (6, 45) = 3.848 P=0.0035
Group:F(2,15)=9.000 P=0.0027
Trainning Day:F(3,45)=19.74 P<0.0001
Swim Path Length (mm)
training days
0.0002
0.0014
0.0001
0.0033
<0.0001
0.0190
VPA
WT
CKO
C
Swim speed (mm/s)
VPA
WT
CKO
D
Swim Path Length (mm)
0.0002
VPA
WT
CKO
