# Supplementary material for: Genetic and pharmacological evidence linking CB1R signaling to hippocampal GABAergic dysfunction in ASD mouse model
Source: Neurotherapeutics. 2026 Sep 18;23(6):e01067. doi: 10.1016/j.neurot.2026.e01067 (PMC13602063; doi:10.1016/j.neurot.2026.e01067)
Supplement: Supplementary file 5 [file mmc1.docx]

**KEY RESOURCES TABLE**

| **Antibodies** | **SOURCE** | **IDENTIFIER** |
| --- | --- | --- |
| Rabbit anti-CB1R | Abcam | Cat#ab259323 |
| Rabbit anti-PSD95 | Proteintech | Cat#20665-1-AP |
| Rabbit anti-GAD67 | Proteintech | Cat#10408-1-AP |
| Rabbit anti-GAD65 | Proteintech | Cat#20746-1-AP |
| Rabbit anti-ABAT | Proteintech | Cat#11349-1-AP |
| Rabbit anti-VGAT | Proteintech | Cat#14471-1AP |
| Rabbit anti-GABARAP | Proteintech | Cat#18723-1-AP |
| Rabbit anti-VGLUT1 | Synaptic Systems | Cat#135302 |
| Rabbit anti-MAP2 | Abcam | Cat#ab183830 |
| Rabbit anti-Neun | Novus | Cat#NBP1-92693PE |
| Mouse anti-Gephyrin | Synaptic Systems | Cat#147111 |
| Mouse anti-Synapsin 1 | Synaptic Systems | Cat#106011 |
| Mouse anti-GAPDH | Proteintech | Cat#60004-1-Ig |

**Oligonucleotides (**PCR genotyping primers)

Sense primer for Cnr1 flox (5’ arm): CATAAGCCTCTGTGCTAATCTTCCTGAT

Antisense primer for Cnr1 flox (5’ arm): GGACAGATAAGACAATCAGTGAAATGG

Sense primer for Cnr1 flox (3’ arm): CATTGGTCCAGAAGTTCCCTCAA

Antisense primer for Cnr1 flox (3’ arm): AGGCTCAAGGAATACACTTATACCCTC

Sense primer for Dlx5/6-Cre (5’-3’ arm): GCGGTCTGGCAGTAAAAACTATC

Antisense primer for Dlx5/6-Cre (5’-3’ arm): GTGAAACAGCATTGCTGTCACTT

**Figure S1. Phenotypic characterization and dose optimization of SA-PEG-DSPE Nanomicelles.**

(A) Body-weight trajectories from postnatal day (P)5 to P40. WT mice exhibited significantly greater body weight than CKO littermates. (Day 5 t_19_=2.738 *P*=0.013, Day 10 t_19_=4.175 *P*=0.001, Day 15 t_19_=3.470 *P*=0.003, Day 20 t_19_=4.648 *P*<0001, Day 25 t_19_=4.863 *P*<0001, Day 30 t_19_=3.003 *P*=0.007, Day 35 t_19_=3.465 *P*=0.003, Day 40 t_19_=3.745 *P*=0.001)

(B) Representative gross brain images showing a slight reduction in brain size in CKO mice relative to WT controls.

(C) Dose-dependent effects of SA-PEG-DSPE@ACPA on CB1R expression in hippocampal tissues of C57BL/6J mice, with maximal upregulation observed at 0.3 mg/kg. (F_3, 8_ = 3.541, *P*=0.068)

(D) Dose-dependent effects of SA-PEG-DSPE@muscimol on GAD67 and GABARAP expression in hippocampal tissues of C57BL/6J mice, with peak expression at 1 mg/kg. (GAD67, F_3, 8_ = 5.580, *P*=0.023. GABARAP, F_3, 8_ = 3.592, *P*=0.066)

All data are presented as means ± SEM, n=3-8 per group; unpaired *t* test, and one-way ANOVA with Sidak post hoc test are used.

**Figure S2 Colocalization of CB1R with GAD67 in GABAergic neurons within hippocampal CA1 and CA3 regions.**

(A and B) Representative double-immunofluorescence staining showing colocalization of CB1R (red) and GAD67 (green) in GABAergic neurons in the hippocampal CA1 (A) and CA3 (B) regions. The upper panels display the overview image, and the lower panels show magnified views of the area indicated by the box in the overview. Scale bars: 100 μm.

(C and D) Fluorescence intensity profiles along the line-scan drawn across the CA1 (C) and CA3 (D) overview, respectively, illustrating the overlapping distribution of CB1R (red curve) and GAD67 (green curve) signals.

**Figure S3. Behavioral data from the three-chamber social test.**

(A) Total distance travelled during the sociability phase of the three-chamber test. One-way ANOVA. (F _2,15_ = 0.891, *P*=0.431)

(B) Total distance travelled during the social preference phase of the three-chamber test. One-way ANOVA. (F 2,15 = 0.098, *P*=0.907)All data are presented as means ± SEM, n= 6 per group; multiple comparisons with Turkey post hoc test.

**Figure S4 Swim speed and swim path length during the training and probe phases of the Morris water maze.**

(A–B) Swim speed (A) and Swim path length (B) of mice during the training days. Two-way ANOVA.

(C–D) Swim speed (C) and swim path length (D) of mice during the probe phase. One-way ANOVA. (Swim path, F_2,15_ = 14.43, *P*=0.0003. Swim speed, F2,15 = 0.1719, *P*=0.8437)

All data are presented as means ± SEM, n= 6 per group; One-way ANOVA multiple comparisons with Turkey post hoc test and two-way ANOVA with Sidak post hoc test.
